# Synthesis, structure and π-expansion of tris(4,5-dehydro-2,3:6,7-dibenzotropone)

**DOI:** 10.3762/bjoc.21.1

**Published:** 2025-01-02

**Authors:** Yongming Xiong, Xue Lin Ma, Shilong Su, Qian Miao

**Affiliations:** 1 Department of Chemistry, The Chinese University of Hong Kong, Shatin, New Territories, Hong Kong, China; 2 State Key Laboratory of Synthetic Chemistry, The Chinese University of Hong Kong, Shatin, New Territories, Hong Kong, China

**Keywords:** carbon schwarzites, polycyclic arenes, Scholl reaction, seven-membered carbocycle, Yamamoto coupling

## Abstract

The polycyclic skeleton of tris(4,5-dehydro-2,3:6,7-dibenzotropone) is a key structural fragment in carbon schwarzites, a theoretical form of negatively curved carbon allotrope. This report presents a new synthesis of this compound using a Ni-mediated Yamamoto coupling reaction and structural analysis of it with X-ray crystallography. Interestingly, it is observed that tris(4,5-dehydro-2,3:6,7-dibenzotropone) crystallized from its solution in hexane resulting in colorless and yellow crystal polymorphs, where it adopts conformations of approximate *C_s_* and *C*_2_ symmetry, respectively. Furthermore, expanding its π-skeleton through the Barton–Kellogg and Scholl reactions led to the successful synthesis of a curved polycyclic arene containing three heptagons and two pentagons.

## Introduction

**Figure 1:**
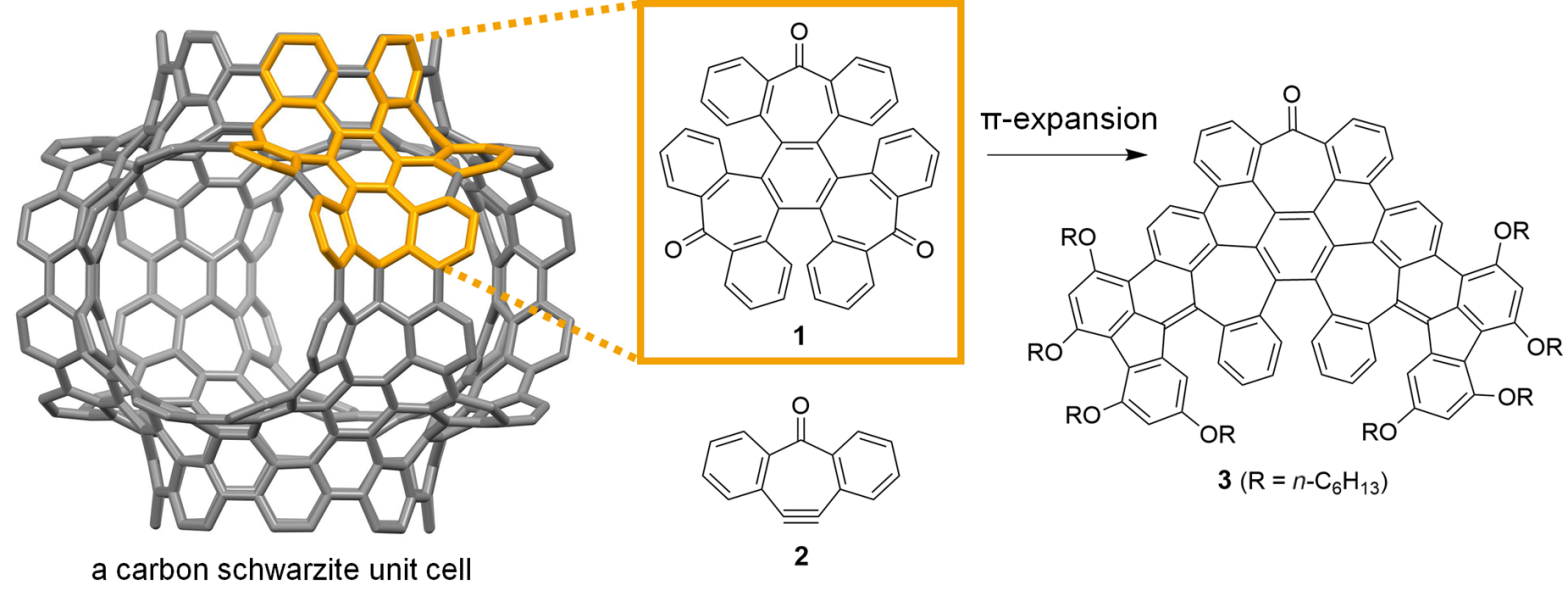
Structures of compounds **1**–**3** and the polycyclic skeleton of **1** as mapped on a carbon schwarzite unit cell.

The title compound (**1** in Figure 1), tris(4,5-dehydro-2,3:6,7-dibenzotropone), receives this name because it can formally result from cyclotrimerization of 4,5-dehydro-2,3:6,7-dibenzotropone (**2** in Figure 1). Similarly, compound **1** was called “a formal trimer of dibenzotropone” or “benzannulated tris-tropone” in literature [1–2], although it should be named as 9*H*,18*H*,27*H*-hexabenzo[*c*,*c*′,*c*′′,*f*,*f*′,*f*′′]benzo[1,2-*a*:3,4-*a*′:5,6-*a*′′]triscycloheptene-9,18,27-trione according to the IUPAC nomenclature. We became interested in compound **1** because its polycyclic skeleton presents a key structural unit in carbon schwarzites, a theoretical form of negatively curved carbon allotrope, as shown in Figure 1.

Carbon allotropes composed solely of sp²-hybridized carbon atoms can form surfaces that range from flat, like a carpet, to curved, resembling shapes such as bowls or saddles. The shape these surfaces take depends on the arrangement of the carbon atoms and is characterized by a geometric property known as curvature. When five-membered rings of carbon atoms are present, they induce a positive curvature, exemplified by fullerenes. Conversely, seven- or eight-membered rings lead to negative curvature, as seen in theoretical carbon structures known as carbon schwarzites or Mackay crystals. These names honor A. L. Mackay and H. A. Schwarz for their pioneering contributions. In 1991, Mackay introduced the idea of negatively curved carbon allotropes by incorporating octagons into the graphitic lattice [3]. Earlier, in the 1880s, Schwarz described triply periodic minimal surfaces, which serve as the topological foundation for what are now known as Mackay crystals. Despite predictions that carbon schwarzites would have intriguing properties for various potential applications [4–5], they have not yet been definitively synthesized. The three-dimensional graphene-like carbons formed in a zeolite-template are the carbon forms that are the closest to carbon schwarzites so far [6–7]. Fragments of carbon schwarzites that retain their key structural characteristics are negatively curved polycyclic arenes [8–9]. These are three-dimensional molecular nanocarbons that include heptagons [10–14], octagons [15–18], or even larger carbocycles. In theory, these fragments can serve as building blocks in a bottom-up approach to constructing carbon schwarzites [19–20]. To validate this concept, we recently showed that polymerizing negatively curved polycyclic arenes produced an amorphous covalent network. This network was able to mimic the structure and function of carbon schwarzites, serving as an anode material in lithium-ion batteries with high capacity [21]. Further exploration of bottom-up approach to carbon schwarzites requires synthesizing of new negatively curved polycyclic arenes and expanding them to lager three-dimensional molecular nanocarbons.

**Scheme 1:**
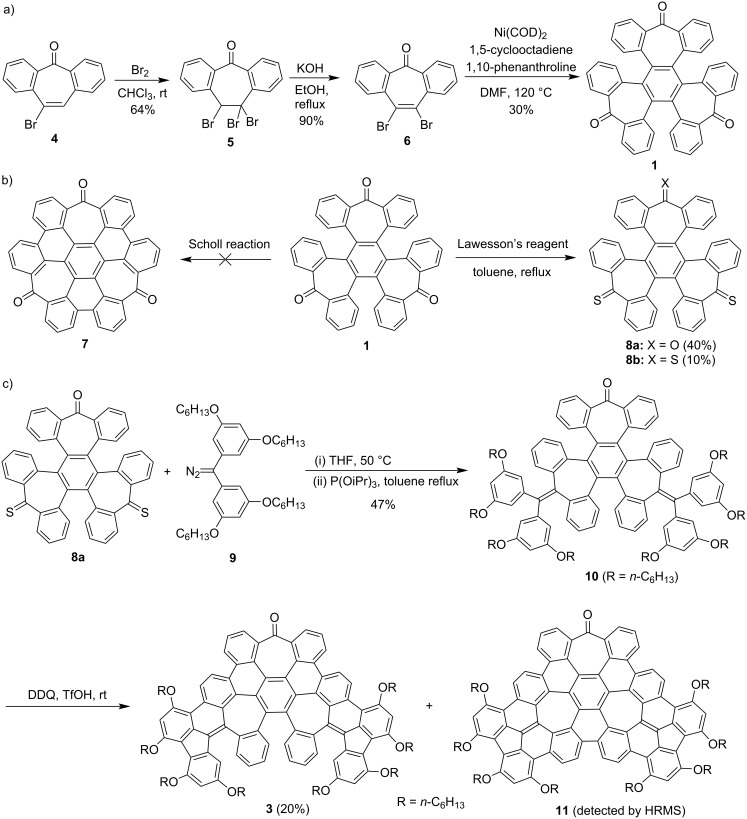
a) Synthesis of **1**; b) reactions of **1**; c) synthesis of **3**.

Compound **1** was recently used as a starting material for the synthesis of nonplanar polycyclic arenes, in particular, molecular models of cubic graphite [22]. It was earlier prepared in Ar matrices [23] or via demetallation of a platinum complex of 4,5-dehydro-2,3:6,7-dibenzotropone [1]. More recently, compound **1** was prepared via Pd-catalyzed cyclotrimerization of 4-bromo-2,3:6,7-dibenzotropone (**4** in Scheme 1) [22]. Herein we report an alternative synthesis of **1** using a Ni-mediated Yamamoto coupling reaction and the simultaneous crystallization of its two different conformers from the same solution. Expanding the π-skeleton of **1** through a Barton–Kellogg reaction followed by a subsequent Scholl reaction resulted in a new polycyclic arene (**3** in Figure 1) featuring three heptagons and two pentagons, with its structure confirmed by X-ray crystallography. This π-expansion approach of compound **1** differs from the method reported by Müllen and co-workers, which involves Ramirez olefination and Suzuki coupling, resulting in the expansion of a seven-membered ring to an eight-membered ring [2].

## Results and Discussion

As shown in Scheme 1a, the synthesis of trione **1** started from the bromination of 4-bromo-2,3:6,7-dibenzotropone (**4**) [24], giving tribromide **5** in a yield of 64%. The subsequent elimination reaction of **5** with KOH afforded dibromide **6** in a yield of 90%. Then, the Ni-mediated Yamamoto coupling reaction of **6** enabled cyclotrimerization to give trione **1** in a yield of 30%. It is worth mentioning that using 1,10-phenanthroline as the ligand in the Yamamoto coupling [25–26] led to a higher yield of compound **1** than using 2,2’-bipyridine. With trione **1** in hands, we explored the Scholl reaction and thionation reaction of it (Scheme 1b) because these reactions can potentially allow π-expansion of **1**. A variety of Scholl reaction conditions, such as AlCl_3_/NaCl, AlCl_3_/CuCl_2_, FeCl_3_, and DDQ/TfOH, were tested. However, these reactions either left the starting material unreacted or resulted in complex mixtures, from which cyclodehydrogenation products, such as compound **7** (Scheme 1b), could not be isolated in a pure form. This can be attributed to the electron-withdrawing carbonyl groups, which make compound **1** unreactive to oxidation. For thionation of the carbonyl groups in **1**, it was treated with three equivalents Lawesson's reagent, affording dithioketone **8a** in a 40% yield together with trithioketone **8b** in a yield of 10%.

To expand the π-skeleton of **1**, compound **8a** was subjected to the Barton–Kellogg reaction with diazo compound **9**, which was synthesized according to the procedures detailed in Supporting Information File 1. In this reaction, the first step of diazo–thioketone coupling occurred at 50 °C in THF, and the second step of desulfurization with triisopropyl phosphite occurred in refluxed toluene, giving diene **10** in a yield of 47%. The Barton–Kellogg reaction with **8b** under similar conditions gave the episulfide intermediate, which, however, could not be desulfurized with triisopropyl phosphite, trimethyl phosphite or triphenylphosphine to give the corresponding triene. The subsequent Scholl reaction of **10** with DDQ and triflic acid at room temperature yielded partially fused nanographene **3** (20%), with formation of six C–C bonds giving four six-membered rings and two five-membered rings. Performing this reaction at a higher temperature led to a lower yield of compound **3** and the formation of byproducts with lower *R*_f_ values on thin-layer chromatography (TLC). When other typical conditions for Scholl reactions, such as FeCl_3_ or DDQ/CH_3_SO_3_H, were employed to treat compound **10**, product **3** was not isolated. Instead, the starting material either remained unreacted or was converted to a complicated mixture of products. It is worth noting that the alkoxy groups in compound **10**, which are positioned *para* or *ortho* to the reacting site, play an important role in the Scholl reaction to form compound **3**. Similar substrates, where the alkoxy groups are replaced by hydrogen atoms or a 4-*tert*-butyl group, did not yield product **3** under similar Scholl reaction conditions. The structure of **3** was confirmed with single crystal X-ray crystallography, as detailed later. In addition, another product with a molecular ion peak of 1695.9115 in the high-resolution mass spectrum (Supporting Information File 1, Figure S6) was isolated in trace amounts. This corresponds to a molecular formula of C_119_H_122_O_9_, which is in agreement with the fully fused product **11** in its protonated form. Unfortunately, clean ^1^H and ^13^C NMR spectra of this product could not be obtained to allow full characterization of this product. Efforts to increase the yield of **11**, such as increasing the amount of DDQ or elevating the reaction temperature in the Scholl reaction of **10**, only resulted in complex mixtures. Further attempts to subject **3** to the Scholl reaction conditions did not yield further cyclized products but led to the decomposition of the starting material. These findings suggest that the fully fused product **11** may have been formed through a different partially cyclized intermediate rather than directly from compound **3**.

**Figure 2:**
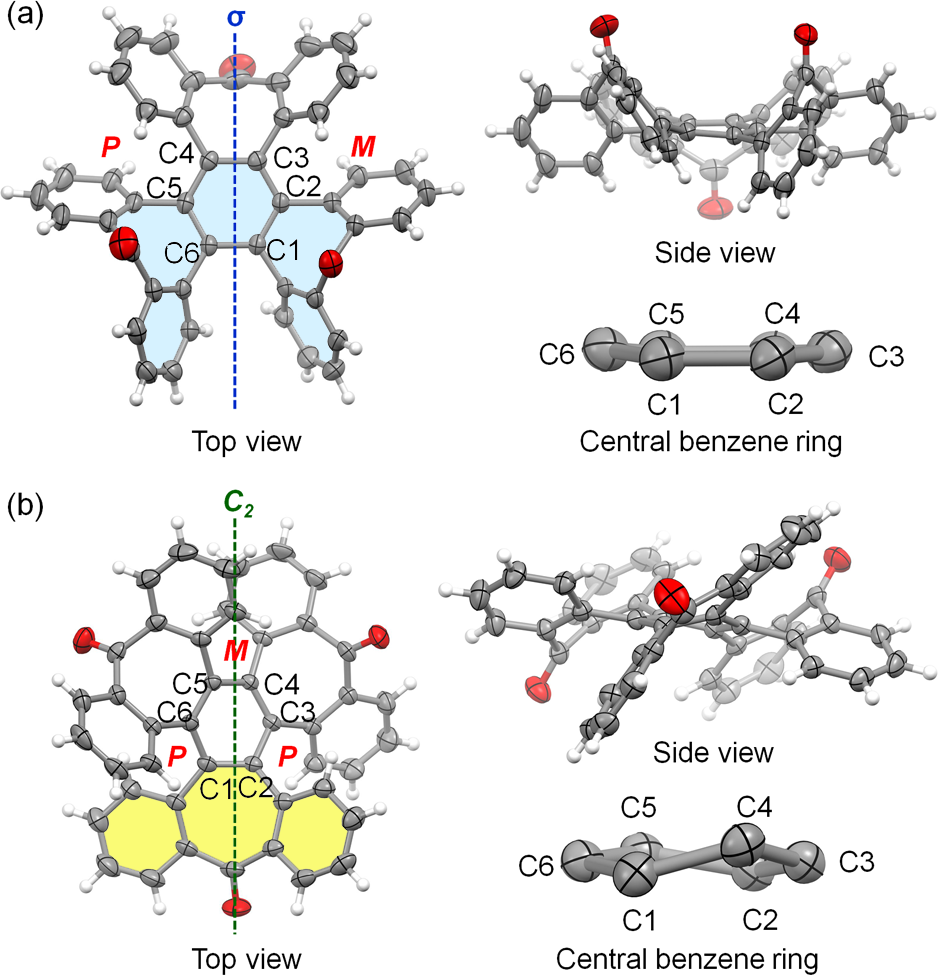
(a) Structures of **1** in the colorless crystal; (b) structures of (*P*,*M*,*P*)-**1** in the yellow crystal. (Carbon atoms are shown as ellipsoids at 50% probability level, in and H atoms are removed for clarification).

Slow evaporation of solvent from a solution of **1** in hexane interestingly resulted in the simultaneous formation of both colorless and yellow crystals from the same solution. X-ray crystallography reveals that in the colorless crystal [27], compound **1** adopts a conformation with approximate *C_s_* symmetry, with the plane of symmetry (σ) shown in the top view in Figure 2a. The structure of *C_s_*-**1** in this crystal is essentially the same as that in the reported crystal structure of **1**·CH_2_Cl_2_ [22]. The side view of compound **1** indicates that two of its carbonyl groups are oriented upwards while the third one points downwards. Compound **1** comprises three [5]helicenoid moieties, each containing three benzene rings and two seven-membered rings. In the colorless crystal, two of these [5]helicenoid moieties display *P* and *M* helix structures respectively, whereas the third moiety (colored in light blue) adopts a structure with approximate plane symmetry, recognized as a transition state for the enantiomerization of helicenes. The central benzene ring of *C_s_*-**1** is essentially flat, exhibiting the largest torsion angle of 8.15° (C4–C5–C6–C1).

Unlike the colorless crystal, the yellow crystal consists of conformers of **1** with approximate *C*_2_ symmetry [27]. They exist as a pair of enantiomers, namely, (*P*,*M*,*P*)-**1** and (*M*,*P*,*M*)-**1**, where *P* and *M* represent the helicity of the three [5]helicenoid moieties. This crystal structure is essentially the same as that reported by Jones earlier [1]. Figure 2b illustrates the structure of (*P*,*M*,*P*)-**1** with the *C*_2_ axis, where one carbonyl group points upward, another downward, and the third one faces forwards. Compound **1** consists of three 2,3:6,7-dibenzotropone moieties. In the yellow crystal, one of these moieties (colored in yellow) is unique due to its less bent seven-membered ring, distinguishing it from the other two dibenzotropone moieties in *C*_2_-**1** and those in *C_s_*-**1**. As a result, *C*_2_-1 in the yellow crystal presents an apparently twisted central benzene ring with large torsion angles: C1–C2–C3–C4 at 19.5°, C3–C4–C5–C6 at 21.8°, and C5–C6–C1–C2 at 21.5°. The crystallization of the two conformers of **1** in different polymorphs suggests the flexibility of its polycyclic skeleton, with both its [5]helicenoid and dibenzotropone moieties capable of flapping with small energy barriers. This is supported by the ^1^H NMR spectrum of compound **1** (Supporting Information File 1, Figure S11), which presents only four different signals due to rapid conformational shifts in solution. Additional evidence of the small energy barrier for conformational change is provided by the earlier report that the NMR spectrum of **1** at −80 °C did not display significant broadening [1].

To better understand the two conformers of **1** found in the crystals, density functional theory (DFT) calculations were carried out using the molecular geometries present in the crystal structures. The results showed that *C*_2_-**1** is more stable than *C_s_*-**1** by 3.85 kcal/mol at the B3LYP/6-311G(d,p) level of DFT. Additionally, *C*_2_-**1** has a smaller gap (3.57 eV) between the highest occupied molecular orbital (HOMO) and the lowest unoccupied molecular orbital (LUMO) compared to *C_s_*-**1** (4.37 eV) as calculated at the B3LYP/6-311++G(d,p) level of DFT. The reduced HOMO–LUMO gap of *C*_2_-**1** can be attributed to the greater conjugation in the essentially flat dibenzotropone moiety. This finding aligns with the fact that the yellow crystal of *C*_2_-**1** absorbs light of a longer wavelength than the colorless crystal of *C_s_*-**1**.

**Figure 3:**
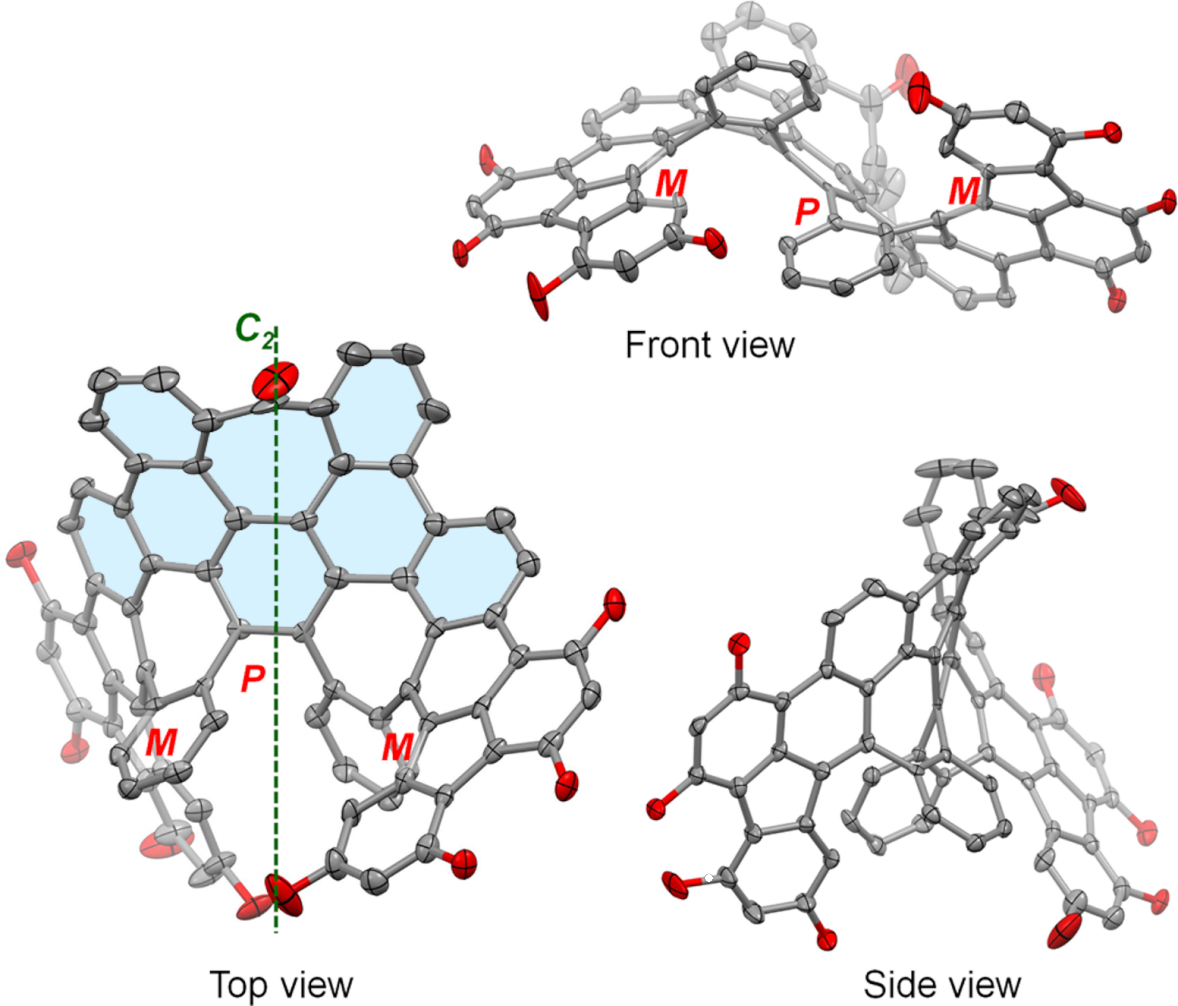
Structure of (*M*,*P*,*M*)-**3** in the crystal of **3**·CH_2_Cl_2_ (carbon and oxygen atoms are shown as grey and red ellipsoid at the level of 50% probability, and hexyl groups and hydrogen atoms are removed for clarity).

Slow evaporation of solvent from a solution of **3** in CH_2_Cl_2_/CH_3_OH resulted in the formation of single crystals suitable for X-ray crystallography [27]. Compound **3** consists of three [5]helicenoid moieties, with two of them containing three benzene rings, one five-membered ring, and one seven-membered ring, and the third one containing three benzene rings and two seven-membered rings. The crystal structure of **3**·CH_2_Cl_2_ reveals that each unit cell contains a pair of enantiomers, (*M*,*P*,*M*)-**3** and (*P*,*M*,*P*)-**3**, co-crystallized with two molecules of CH_2_Cl_2_. Here *P* and *M* represent the helicity of the three [5]helicenoid moieties. The geometry of **3** deviates from ideal *C*_2_ symmetry, as the light blue moiety of **3** (top view in Figure 3) is shaped like a saddle. However, the ^1^H NMR spectrum of **3** shows only 12 different signals in the aromatic region, indicating a two-fold symmetry in the polycyclic skeleton of **3**. This indicates that the polycyclic skeleton of **3** is flexible, similar to that of **1**. In the crystal, the neighbouring enantiomers of **3** show minimal π-overlap with each other and a large π to π distance of 3.68 Å between terminal benzene rings.

**Figure 4:**
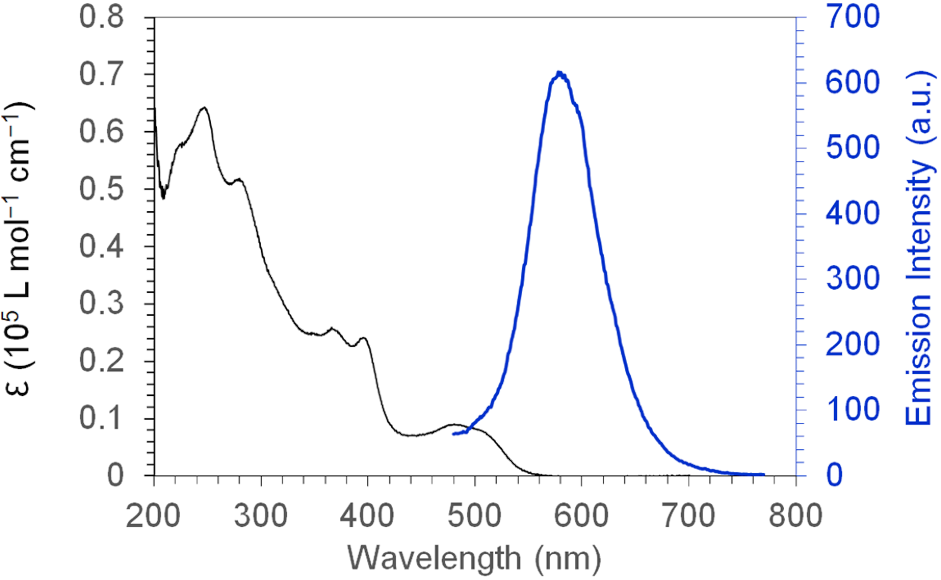
UV–vis absorption spectrum (black line) and emission spectrum (blue line, excited at 400 nm) of compound **3** in cyclohexane (1 × 10^−5^ mol/L).

Compound **3** forms orange solution in common organic solvents, and its solution in cyclohexane exhibits very weak orange photoluminescence with a quantum yield as low as 3.9 × 10^−4^ upon excitation at 400 nm. Such a low photoluminescence quantum yield may be attributed to the conformational motions of the helicene moieties in **3**, which consume the energy of the excited state. Figure 4 shows the UV–vis absorption spectrum of **3** in cyclohexane with the absorption edge at 561 nm and its emission spectrum with a peak at 580 nm. In the test windows of cyclic voltammetry (Supporting Information File 1, Figure S1), **3** exhibits one reversible oxidation wave and one quasi-reversible oxidation wave with half-wave potentials of 0.40 V and 0.88 V, respectively, versus ferrocenium/ferrocene (Fc^+^/Fc). The HOMO energy level is estimated from the first oxidation peak to be −5.5 eV [28], which is consistent with the DFT-calculated HOMO level (−5.47 eV).

## Conclusion

In conclusion, we developed a new synthesis of tris(4,5-dehydro-2,3:6,7-dibenzotropone) (**1**) through a Ni-mediated Yamamoto coupling reaction. Upon crystallization from the same solution in hexane, this compound yielded colorless and yellow crystal polymorphs, adopting conformations of approximate *C_s_* and *C*_2_ symmetry, respectively. Furthermore, the expansion of the π-skeleton of **1** through the Barton–Kellogg and Scholl reactions enabled the synthesis of compound **3**, whose curved polycyclic skeleton containing three heptagons and two pentagons was identified with X-ray crystallography.

## Supporting Information

File 1 Experimental details, characterization data, and spectra.

File 2 CIF-files of compounds **1** and **3**.

## Data Availability

All data that supports the findings of this study is available in the published article and/or the supporting information of this article.
