# Supplementary material for: Synthesis, structure and π-expansion of tris(4,5-dehydro-2,3:6,7-dibenzotropone)
Source: Beilstein J Org Chem. 2025 Jan 2;21:1–7. doi: 10.3762/bjoc.21.1 (PMC11702293; doi:10.3762/bjoc.21.1)
Supplement: File 1 — Experimental details, characterization data, and spectra. [file Beilstein_J_Org_Chem-21-01-s001.pdf]

BEILSTEIN JOURNAL OF ORGANIC CHEMISTRY

Supporting Information

for

Synthesis, structure and π-expansion of tris(4,5-dehydro-2,3:6,7-dibenzotropone)

Yongming Xiong, Xue Lin Ma, Shilong Su and Qian Miao

Beilstein J. Org. Chem. **2025,** *21,* 1–7. doi:10.3762/bjoc.21.1

Experimental details, characterization data, and spectra

License and Terms: This is a supporting information file under the terms of the Creative Commons Attribution License (https://creativecommons.org/licenses/by/4.0). Please note that the reuse, redistribution and reproduction in particular requires that the author(s) and source are credited and that individual graphics may be subject to special legal provisions.
The license is subject to the *Beilstein Journal of Organic Chemistry* terms and conditions: (https://www.beilstein-journals.org/bjoc/terms)

Table of Contents

1. Synthesis
2. X-ray crystallography
3. Cyclic voltammetry
4. Determination of photoluminescence quantum yield
5. Density functional theory (DFT) calculations
6. High resolution mass spectra
7. NMR spectra
8. References

1. Synthesis

General: The reagents and starting materials employed were commercially available and used without any further purification or made following reported methods as indicated. Anhydrous and O_2-free THF and Et_2O were purified by an Innovative Technology Solvent Purification System PS-MD-4. Gel permeation chromatography (GPC) were performed on a JAI LC-9160 II NEXT automatic recycling preparative HPLC system with a UV–vis detector. NMR spectra were recorded on a Bruker AVANCE III 400 MHz spectrometer (^{1}H NMR: 400 MHz, ^{13}C NMR: 100 MHz). Abbreviations: s = singlet, d = doublet, t = triplet, m = multiplet. Chemical shift values (δ) are expressed in parts per million using residual solvent protons (^{1}H NMR, δH = 2.50 for DMSO-d_6, δH = 5.32 for CD_2Cl_2, δH = 7.26 for $CDCl_3$, ^{13}C NMR, δC = 77.16 for $CDCl_3$, δC = 39.52 for DMSO-d_6) as internal standard. Mass spectra were recorded on Therno Finnigan MAT 95 XL spectrometer or a Bruker Autoflex speed MALDI–TOF spectrometer. UV-vis absorption spectra were recorded on a Shimadzu UV-3600 Plus UV-VIS-NIR Spectrophotometer. Fluorescence spectra were taken on a HITACHI F-4500 spectrofluorometer. Unless otherwise noted, melting points, without correction, were measured using a Nikon Polarized Light Microscope ECLIPSE 50i POL equipped with an INTEC HCS302 heating stage

4-Bromo-2,3:6,7-dibenzotropone (**4**) was synthesized according to the reported procedure.[1]

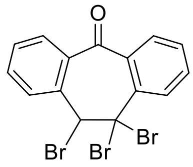

Compound 5: To a solution of **4** (6.74 g, 24.4 mmol) in 50 mL of CH_2Cl_2 was added Br_2 (1.3 mL, 24.4 mmol) in ambient air. The resulting solution was stirred at room temperature for 2 days, and then quenched with a saturated aqueous solution of Na_2SO_3. The reaction mixture was then extracted with CH_2Cl_2, and the organic layer was dried over anhydrous $MgSO_4$. The resulting solution was concentrated under reduced pressure to afford a white crude product containing **4** and **5**. The obtained white solid was dissolved in a small amount of CH_2Cl_2 to afford a pale yellow solution. The resulting CH_2Cl_2 solution was added a large amount of hexane and then subjected to ultrasonication for several minutes to afford a white precipitate. After filtration, 4.0 g of **5** was obtained as white solid. The filtrate containing **4** and **5** was concentrated and then subjected to the above bromination process again for one day. Repeating the precipitation process afforded 3.0 g of **5**. The total yield of **5** is 64%. mp: 140-142°C. ^{1}H NMR ($CDCl_3$, 400MHz) δ (ppm): 8.32 (d, J = 8 Hz, 1H), 7.98 (dd, J_1 = 7.9 Hz, J_2 = 1 Hz, 1H), 7.79 (d, J = 7.2 Hz, 1H), 7.65, (dd, J_1=7.7 Hz, J_2=, 7.32 Hz, 1H), 7.55 (dd, J_1=7.4 Hz, J_2=, 7.4 Hz, 1H), 7.49 (m, 2H), 7.43 (d, J = 7.4 Hz, 1H), 6.12 (s, 1H). ^{13}C NMR ($CDCl_3$, 400MHz) δ (ppm): 193.3, 140.0, 139.4, 136.8, 134.2, 134.1, 133.3, 132.2, 130.3, 130.2, 130.0, 129.4, 67.4, 64.7. HRMS (APCI): calcd. for $C_{15}H_9Br_3O$ ($[M+H]^+$): 444.82562 ; found: 444.82567.

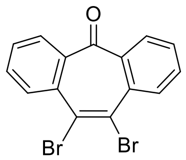

Compound **6**: To a suspension of **5** (1.33 g, 3 mmol) in ethanol (12 mL) was added a solution of KOH (505 mg, 9 mmol) in ethanol (10 mL) in ambient air. The resulting mixture was heated to reflux for 2 hours. After cooled to room temperature, the resulting suspension was filtered and the solid residue was washed with water and methanol. After dried in vacuum, compound **6** (984 mg, 90%) was obtained as pale-yellow solid. mp: 135-136 °C. ^{1}H NMR ($CDCl_3$, 400MHz) δ (ppm): 7.98 (dd, J_1 = 7.9 Hz, J_2 = 1 Hz, 2H). 7.57 (dd, J_1 = 7.5 Hz, J_2 = 1.4 Hz, 2H), 7.52 (m, 2H), 7.47 (m, 2H); ^{13}C NMR ($CDCl_3$, 400MHz) δ (ppm): 196.2, 141.1, 133.8, 132.2, 131.1, 130.0, 126.9, 126.3. HRMS (APCI): calcd. for $C_{15}H_8Br_2O$ ($[M+H]^+$): 364.89948; found: 364.89963.

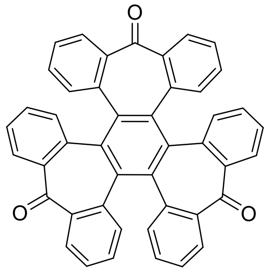

Compound **1**: Under an atmosphere of N_2, a mixture of 1,10-phenanthroline (360 mg, 2 mmol), 1,5-cyclooctadiene (COD; 0.74 mL, 6 mmol) and bis(1,5-cyclooctadiene)nickel(0) (275 mg, 1 mmol) in anhydrous and degassed DMF (4 mL) was stirred at 80 °C for 30 min. A solution of **6** (364 mg, 1 mmol) in anhydrous and degassed DMF (6 mL) was added dropwise to the mixture, and the mixture was then stirred at 120 °C for 2 days. After diluted with water, the mixture was extracted with ethyl acetate and the organic layer was washed with brine, dried with anhydrous $MgSO_4$ and concentrated under reduced pressure. The crude product was purified by silica gel chromatography (eluent:hexane/dichloromethane/ethyl acetate=4/1/0.5) to afford **1** (61 mg, 30%) as a yellow solid. mp: 178-179°C. ^{1}H NMR ($CDCl_3$, 400MHz) δ (ppm): 7.36 (d, J= 7.5 Hz, 6H), 7.13 (m, 6H), 6.94-6.88 (m, 12H). ^{13}C NMR ($CDCl_3$, 400MHz) δ (ppm): 198.5, 146.4, 137.3, 133.9, 132.6, 128.8, 127.8, 124.4. HRMS (APCI): calcd. for $C_{45}H_{24}O_3$ ($[M+H]^+$): 613.17982; found: 613.17989.

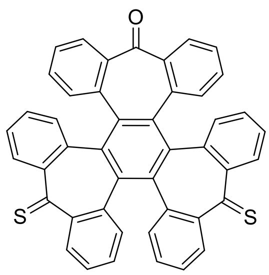

8a

and

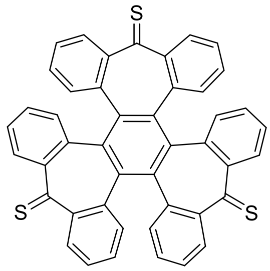

8b

Compound **8a/b**: A 50 mL Schlenck flask was charged with **1** (57 mg, 0.093 mmol) and toluene

(10 mL) under an atmosphere of N_2. To the stirred solution of **1**, Lawesson's reagent (113 mg, 0.28 mmol) was added under a N_2 flow. The resulting suspension was refluxed for 24 h and then quenched with an aqueous solution of NaClO. The mixture was extracted with ethyl acetate and the organic phase was separated, dried with anhydrous $MgSO_4$, concentrated under reduced pressure. The crude product was purified with silica gel chromatography (eluent: hexane/dichloromethane/diethyl ether/triethylamine=4/1/0.5/0.01) to give di-thionated product **8a** (23 mg, 40%) as a gray powder and tri-thionated product **8b** (6 mg, 10%) as a gray powder.
Compound 8a: mp: decomposition from 200°C. ^{1}H NMR ($CDCl_3$, 400MHz) δ (ppm): 7.37 (m, 6H), 7.17 (m, 6H), 6.95 (m, 12H).^{13}C NMR ($CDCl_3$, 400MHz) δ (ppm): 245.2, 198.4, 156.6, 146.2, 137.3, 137.3, 134.1, 132.6, 132.2, 131.8, 131.3, 131.2, 128.8, 127.8, 127.7, 127.6, 124.6, 124.5, 124.4. HRMS (APCI): calcd. for $C_{45}H_{24}OS_2$ ($[M+H]^+$): 645.13413; found: 645.13411.
Comopound 8b: mp: decomposition from 200°C. ^{1}H NMR ($CDCl_3$, 500MHz) δ (ppm): 7.29 (d, J =7.7 Hz, 6H), 7.09 (m, 6H), 6.9 (m, 12H). ^{13}C NMR ($CDCl_3$, 500MHz) δ (ppm): 245.3, 156.7, 136.9, 131.9, 131.1, 127.6, 127.6, 124.3. HRMS (APCI): calcd. for $C_{45}H_{24}S_3$ ($[M+H]^+$): 661.11129; found: 661.11161.

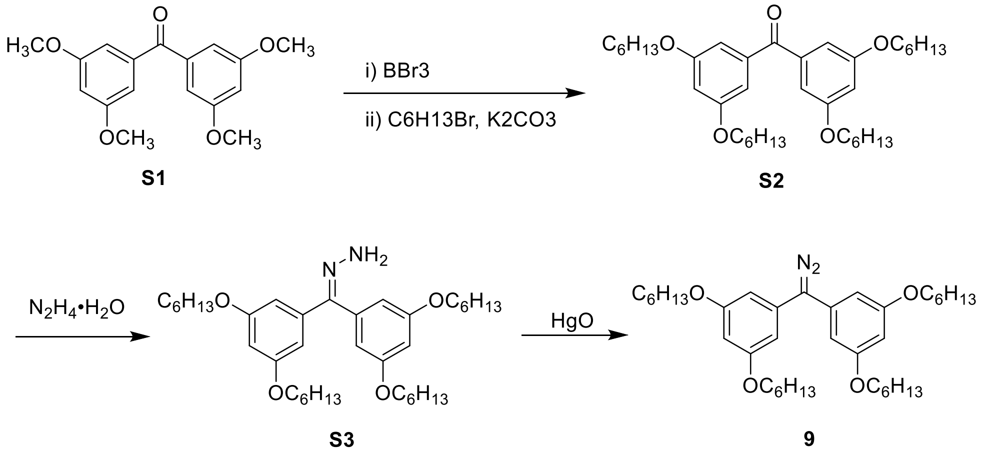

Compound **S2**: Under an atmosphere of N_2, **S1**[2] (906 mg, 3 mmol) was dissolved in anhydrous CH_2Cl_2 (30 mL). To the resulting solution was added BBr_3 (18 mL, 1 M in CH_2Cl_2) and the resulting solution was stirred at room temperature overnight. After the reaction was completed, the reaction mixture was poured into a beaker charged with ice and then extracted with ethyl acetate. The combined organic phase was dried with $MgSO_4$ and then concentrated under reduced pressure. The residue and K_2CO_3 powder (2.07 g, 15 mmol) were loaded in a Schlenck flask (100 mL) and the system was purged with N_2 three times. 1-bromohexane (2.5g, 15 mmol) and DMF (30 mL) were added via syringe. The suspension was stirred at 80 °C for 2 days. After cooling down to room temperature, the resulting mixture was quenched with water, extracted with ethyl acetate, dried with anhydrous $MgSO_4$. The organic phase was concentrated under reduced pressure and the crude product was purified by column chromatography on silica gel with hexane/diethyl ether as eluent to give **S2** (800 mg, 46%) as oil. ^{1}H NMR ($CDCl_3$, 400MHz) δ (ppm): 6.89 (d, J = 2.2 Hz, 4H), 6.66 (t, J = 2.2 Hz, 2H), 3.98 (t, J = 6.5 Hz, 8H), 1.80 (m, 8H), 1.46 (m, 8H), 1.34 (m, 16H), 0.91 (m, 12H). ^{13}C NMR ($CDCl_3$, 400MHz) δ

(ppm): 196.1, 160.0, 139.4, 108.3, 105.6, 68.3, 31.6, 29.2, 25.7, 22.6, 14.0. HRMS (APCI): calcd. for $C_{37}H_{58}O_5$ ($[M+H]^+$): 583.43570; found: 583.43577.

Compound S3: A 25 mL round bottom flask was charged with **S2** (1.16 g, 2 mmol), ethanol (4 mL), $N_2H_4{\cdot}H_2O$ (2 mL), acetic acid (1 mL) and then heated to reflux for 1 day in ambient air. After cooling down to room temperature, the resulting mixture was quenched with $NaHCO_3$ (aq), extracted with ethyl acetate. The organic phase was separated, dried with anhydrous $MgSO_4$, concentrated under reduced pressure. The crude product of **S3** was NMR pure and was directly used for next step without purification. ^{1}H NMR ($CDCl_3$, 400MHz) δ (ppm): 6.66 (d, *J* = 2.2 Hz, 2H), 6.51 (t, *J* = 2.2 Hz, 1H), 6.40 (t, *J* = 2.2 Hz, 1H), 6.34 (d, *J* = 2.2 Hz, 2H), 5.46 (s, 2H), 3.94 (m, 8H), 1.80 (m, 8H), 1.45 (m, 8H), 1.34 (m, 16H), 0.92 (m, 12H). ^{13}C NMR ($CDCl_3$, 400MHz) δ (ppm): 161.3, 160.2, 149.3, 140.1, 134.8, 106.6, 105.2, 102.0, 101.4, 68.3, 31.7, 29.3, 29.3, 25.8, 25.8, 22.7, 14.2. HRMS (APCI): calcd. for $C_{37}H_{60}N_2O_4$ ($[M+H]^+$): 597.46258; found: 597.46239.

Compound 9: A 25 mL round bottom flask connected with a drying tube was charged with **S3** (120 mg, 0.2 mmol), Et_2O (4 mL). Na_2SO_4 (120 mg, 0.8 mmol), HgO (105 mg, 0.4 mmol) were then added to the reaction mixture. To this suspension was added 1 mL of a freshly prepared concentrated solution of KOH in ethanol. The reaction mixture was stirred at room temperature for 12 hours. The resulting suspension was filtered with celite and the solid residue was washed with Et_2O. The red filtrate was concentrated under reduced pressure. The crude product of **9** was directly used for next step immediately without further purification and characterization.

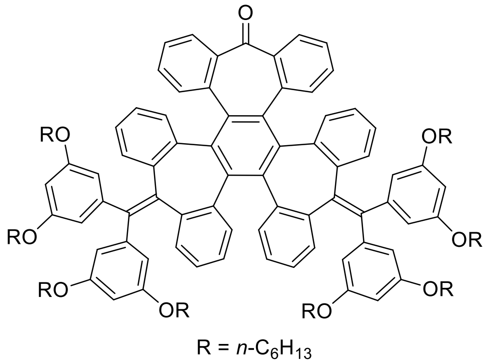

Compound **10**: A 25 mL Schlenk flask was charged with **8a** (30 mg, 0.46 mmol) and dry THF (4 mL) under an atmosphere of N_2. The diazo compounds **9** (0.2 mmol) prepared from the last step was dissolved in Et_2O (4 mL) and the prepared red solution was added to the flask via syringe. The reaction mixture was stirred at 50 °C overnight under exclusion of light. After cooling down to room temperature, solvent was removed under reduced pressure. The residue was dissolved in dry toluene (10 mL) under an atmosphere of N_2. To the resulting solution, P(O-iPr)$_3$ (0.2 mL) was added and the reaction mixture was heated to reflux overnight. After cooling down to room temperature, solvent was removed under reduced pressure and the crude product was purified with silica gel chromatography (eluent: hexane/dichloromethane/diethyl ether=12/1/0.5) to give **10** (37 mg, 47%) as a sticky solid. ^{1}H NMR ($CDCl_3$, 400MHz) δ (ppm):

7.18-7.15 (m, 4H), 7.13-7.11 (d, J = 8.0 Hz, 2H), 7.04-7.01 (m, 4H), 6.96-6.92 (m, 4H), 6.84-6.76 (m, 4H), 6.72 (t, J = 7.4 Hz, 2H), 6.44 (s, 4H), 6.28-6.24 (m, 8H), 6.18 (s, 2H), 6.04 (d, J = 8Hz, 2H), 3.75-3.69 (m, 4H), 3.67-3.62 (m, 8H), 3.56-3.50 (m, 4H), 1.65-1.62 (m, 8H), 1.43-1.25 (m, 36H), 1.14-1.08 (m,16H), 0.88-0.78 (m, 28H). ^{13}C NMR ($CDCl_3$, 500MHz) δ (ppm): 198.4, 159.8, 159.6, 148.2, 147.7, 147.4, 142.8, 141.3, 139.9, 138.5, 138.1, 137.1, 134.9, 134.6, 134.5, 134.3, 132.8, 132.8, 132.4, 127.8, 127.1, 126.8, 126.6, 126.3, 126.1, 124.3, 123.9, 123.6, 108.5, 108.3, 100.5, 100.1, 68.0, 67.8, 31.7, 31.6, 29.3, 29.2, 25.8, 25.7, 22.7, 22.6, 14.2, 14.2. HRMS (APCI): calcd. for $C_{119}H_{140}O_9$ ([M+H]$^+$): 1714.05701; found: 1714.05851.

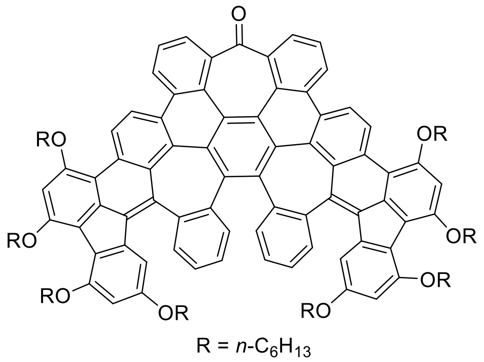

Compound **3**: To a stirred solution of **10** (37 mg, 0.022 mmol) and DDQ (49 mg, 0.22 mmol) in 22 mL of anhydrous CH_2Cl_2 under N_2 was added 0.5 mL of trifluoromethanesulfonic acid. The mixture was stirred for 1.5 hours at room temperature. The reaction was quenched with an aqueous solution of $NaHCO_3$ and then extracted with CH_2Cl_2. The organic layer was washed with brine, dried with anhydrous Na_2SO_4, and concentrated under reduced pressure. The crude product was purified by column chromatography on silica gel with hexane/CH_2Cl_2 = 1/1 as eluent to afford a crude product, which was further purified by preparative thin layer chromatography (PTLC) with hexane/CH_2Cl_2 = 2/1 as eluent to afford compound **3** (7.5mg, 20%) as orange solid. mp: 189-191°C. ^{1}H NMR ($CDCl_3$, 400MHz) δ (ppm): 9.60 (d, J = 9.2 Hz, 2H), 8.53 (d, J = 6.8 Hz, 2H), 8.21 (d, J = 9.4 Hz, 4H), 8.12 (d, J = 8.5 Hz, 2H), 7.67 (t, J = 7.6 Hz, 2H), 7.59 (d, J = 8.0 Hz, 2H), 7.20 (d, J = 7.6 Hz, 2H), 7.15 (t, J = 7.4 Hz, 2H), 7.09 (t, J = 7.4 Hz , 2H), 6.98 (d, J = 1.8 Hz, 2H), 6.84 (s, 2H), 6.57 (d, J = 1.8 Hz, 2H), 4.37 (m, 6H), 4.23 (m, 6H), 3.92 (m, 2H), 3.79 (m, 2H), 2.10 (m, 12H), 1.78 (m,4H), 1.67 (m, 4H). 1.63 (m, 16H), 1.41 (m, 32H), 0.96 (m, 24H). ^{13}C NMR ($CDCl_3$, 400MHz) δ (ppm): 193.8, 159.1, 158.4, 154.8, 153.9, 141.2, 140.7, 139.8, 139.4, 136.5, 136.2, 135.6, 135.5, 134.3, 133.9, 133.7, 131.5, 129.8, 128.8, 128.5, 128.2, 127.6, 127.3, 127.2, 127.0, 127.0, 126.7, 126.3, 124.4, 122.3, 115.5, 113.1, 104.4, 100.9, 100.5, 70.6, 68.7, 68.1, 32.0, 31.9, 31.8, 29.8, 29.6, 29.5, 26.2, 26.1, 25.9, 22.8, 22.8, 22.7, 14.2. HRMS (APCI): calcd. for $C_{119}H_{140}O_9$ ([M+H]$^+$): 1701.96311; found: 1701.96493.

2. X-ray crystallography

X-ray crystallography data were collected on a Bruker AXS Kappa ApexII Duo Diffractometer.

Table **S1**. Crystal data and structure refinement for *C*$_s$-**1**.

Empirical formula	$C_{45}H_{24}O_3$	
Formula weight	612.64	
Temperature	297(2) K	
Wavelength	0.71073 Å	
Crystal system	Monoclinic	
Space group	C2/c	
Unit cell dimensions	a = 28.0213(7) Å	α = 90°
	b = 11.2681(3) Å	β = 97.4740(10)°
	c = 22.2666(6) Å	γ = 90°
Volume	6970.9(3) $Å^3$	
Z	8	
Density (calculated)	1.168 Mg/m^3	
Absorption coefficient	0.072 mm^{-1}	
F(000)	2544	
Crystal size	0.500 x 0.400 x 0.300 mm^3	
Theta range for data collection	2.116 to 25.245°.	
Index ranges	-33<=h<=33, -13<=k<=13, -26<=l<=26	
Reflections collected	38790	
Independent reflections	6285 [R(int) = 0.0314]	
Completeness to theta = 25.242°	99.5 %	
Absorption correction	multi-scan	
Max. and min. transmission	0.7456 and 0.6884	
Refinement method	Full-matrix least-squares on F^2	
Data / restraints / parameters	6285 / 0 / 433	
Goodness-of-fit on F^2	1.072	
Final R indices [I>2sigma(I)]	R1 = 0.0402, wR2 = 0.1001	
R indices (all data)	R1 = 0.0502, wR2 = 0.1065	
Extinction coefficient	n/a	
Largest diff. peak and hole	0.155 and -0.159 e.$Å^{-3}$	

Table **S2**. Crystal data and structure refinement for C_2-**1**.

Empirical formula	C45 H24 O3	
Formula weight	612.64	
Temperature	296(2) K	
Wavelength	0.71073 Å	
Crystal system	Triclinic	
Space group	P-1	
Unit cell dimensions	a = 11.2704(10) Å	α = 111.782(3)°
	b = 11.8263(12) Å	β = 106.331(2)°
	c = 13.4751(11) Å	γ = 98.616(3)°
Volume	1534.1(2) $Å^3$	
Z	2	
Density (calculated)	1.326 Mg/m^3	
Absorption coefficient	0.082 mm^{-1}	
F(000)	636	
Crystal size	0.500 x 0.400 x 0.300 mm^3	
Theta range for data collection	2.331 to 25.250°.	
Index ranges	-13<=h<=13, -14<=k<=14, -16<=l<=16	
Reflections collected	40355	
Independent reflections	5531 [R(int) = 0.1670]	
Completeness to theta = 25.242°	99.7 %	
Absorption correction	multi-scan	
Max. and min. transmission	0.7456 and 0.7035	
Refinement method	Full-matrix least-squares on F^2	
Data / restraints / parameters	5531 / 0 / 433	
Goodness-of-fit on F^2	1.071	
Final R indices [I>2sigma(I)]	R1 = 0.0586, wR2 = 0.1533	
R indices (all data)	R1 = 0.0694, wR2 = 0.1646	
Extinction coefficient	n/a	
Largest diff. peak and hole	0.265 and -0.268 e.$Å^{-3}$	

Table S3 Crystal data and structure refinement for **3**.

Empirical formula	C120 H124 Cl2 O9	
Formula weight	1781.08	
Temperature	173(2) K	
Wavelength	0.71073 Å	
Crystal system	Triclinic	
Space group	P-1	
Unit cell dimensions	a = 8.6131(18) Å	□= 81.365(5)°.
	b = 17.552(4) Å	□= 85.205(5)°.
	c = 33.100(7) Å	□ = 88.771(5)°.
Volume	4929.7(18) $Å^3$	
Z	2	
Density (calculated)	1.200 Mg/m^3	
Absorption coefficient	0.126 mm^{-1}	
F(000)	1900	
Crystal size	0.500 x 0.300 x 0.200 mm^3	
Theta range for data collection	2.057 to 25.250°.	
Index ranges	-10<=h<=10, -21<=k<=21, -39<=l<=39	
Reflections collected	121437	
Independent reflections	17834 [R(int) = 0.1667]	
Completeness to theta = 25.242°	99.9 %	
Absorption correction	multi-scan	
Max. and min. transmission	0.7456 and 0.6160	
Refinement method	Full-matrix least-squares on F^2	
Data / restraints / parameters	17834 / 112 / 1180	
Goodness-of-fit on F^2	1.073	
Final R indices [I>2sigma(I)]	R1 = 0.1487, wR2 = 0.3561	
R indices (all data)	R1 = 0.2170, wR2 = 0.3943	
Extinction coefficient	n/a	
Largest diff. peak and hole	1.766 and -0.747 e.$Å^{-3}$	

3. Cyclic voltammetry

The cyclic voltammetry was performed in a solution of CH_2Cl_2 with 0.1M Bu_4NPF_6 as the supporting electrolyte, at a scan rate of $50 mVs^{-1}$. Ferrocene/ferrocenium was used as the internal standard. Potentials were referenced to ferrocenium/ferrocene ($FeCp_2^+/FeCp_2^0$).

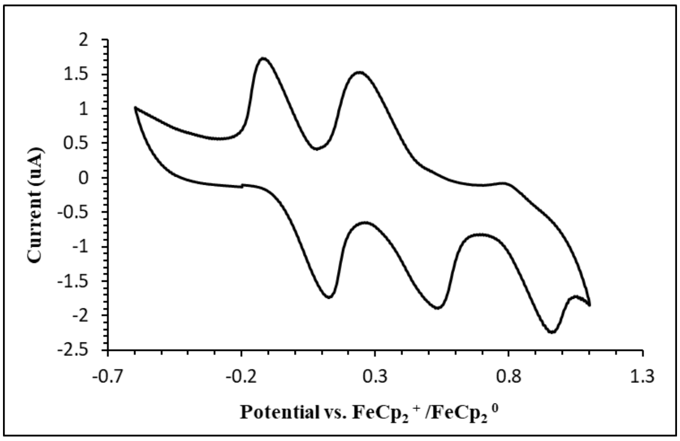

Figure S1 Cyclic voltammogram of **3**.

4. Determination of photoluminescence quantum yield

The photoluminescence quantum yield (Φ) of compound **3** was determined by comparing the photoluminescence (PL) integrated intensities (area) and absorbance intensities with perylene as reference. The quantum yield was calculated by using the following equation:

$$\Phi_{sample} = \left(\frac{F_{sample}}{F_{ref}}\right)\left(\frac{A_{ref}}{A_{sample}}\right)\left(\frac{\eta_{sampe}}{\eta_{ref}}\right)^2 \Phi_{ref}$$

Where F is the integrated intensities (area under emission peak), A is the absorbance, η is the refractive index and Φ is the quantum yield, the subscript "ref" refers to reference. Both perylene and **3** was dissolved in cyclohexane ($\eta = 1.43$). Absorbance values were kept below 0.05 at the excitation wavelength in order to minimize reabsorption effects.

Table S3 Data for determination of quantum yield

	Integrated Intensity (F)	Absorbance at 400 nm (A)	Refractive Index (η)	Quantum yield (Φ)
**Perylene** in cyclohexane	230711	0.012	1.43	0.94 [3]
**3** in cyclohexane	190	0.024	1.43	3.9×10^{-4}

5. Density function theory (DFT) calculation

All the calculations were done using Gaussian 16 Program[4]. **3** were simplified to model molecule **3'**, which has smaller methyl groups replacing hexyl groups. **3'** were optimized at B3LYP/6-31G(d,p) level. Vibrational analysis for C_2-**1** and C_s-**1** were conducted at B3LYP/6-311G(d,p) level based on molecular geometries from crystal structures. All the highest occupied molecular orbitals (HOMO) and lowest unoccupied molecular orbitals (LUMO) were calculated at B3LYP/6-311++G(d,p) level.

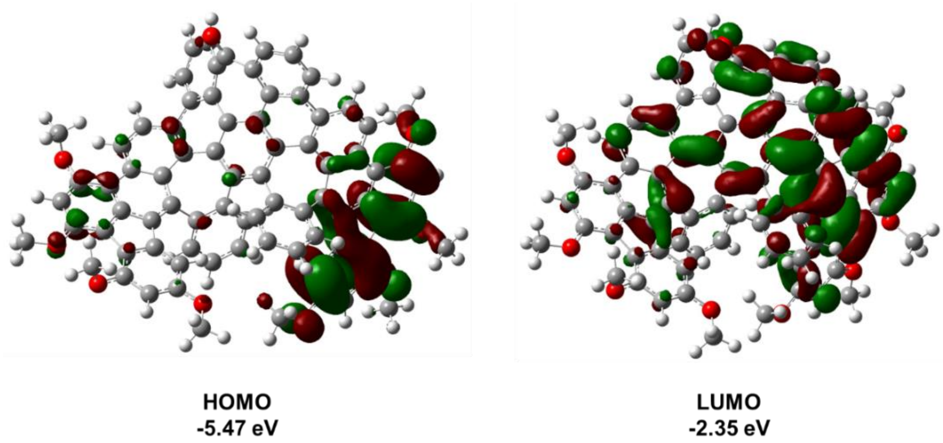

Figure S2 HOMO and LUMO of **3’**.

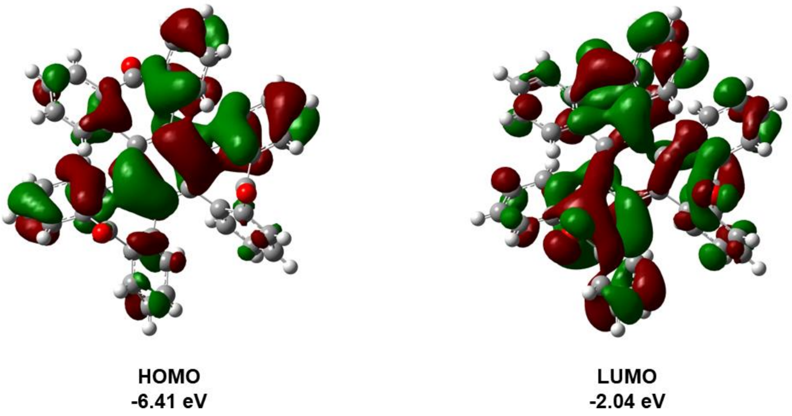

Figure S3 HOMO and LUMO of C_s-**1**.

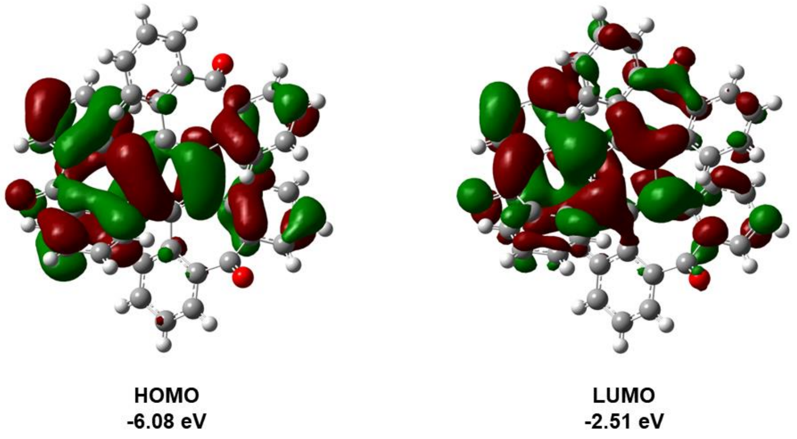

Figure S4 HOMO and LUMO of C_2-**1**.

6. High resolution mass spectra

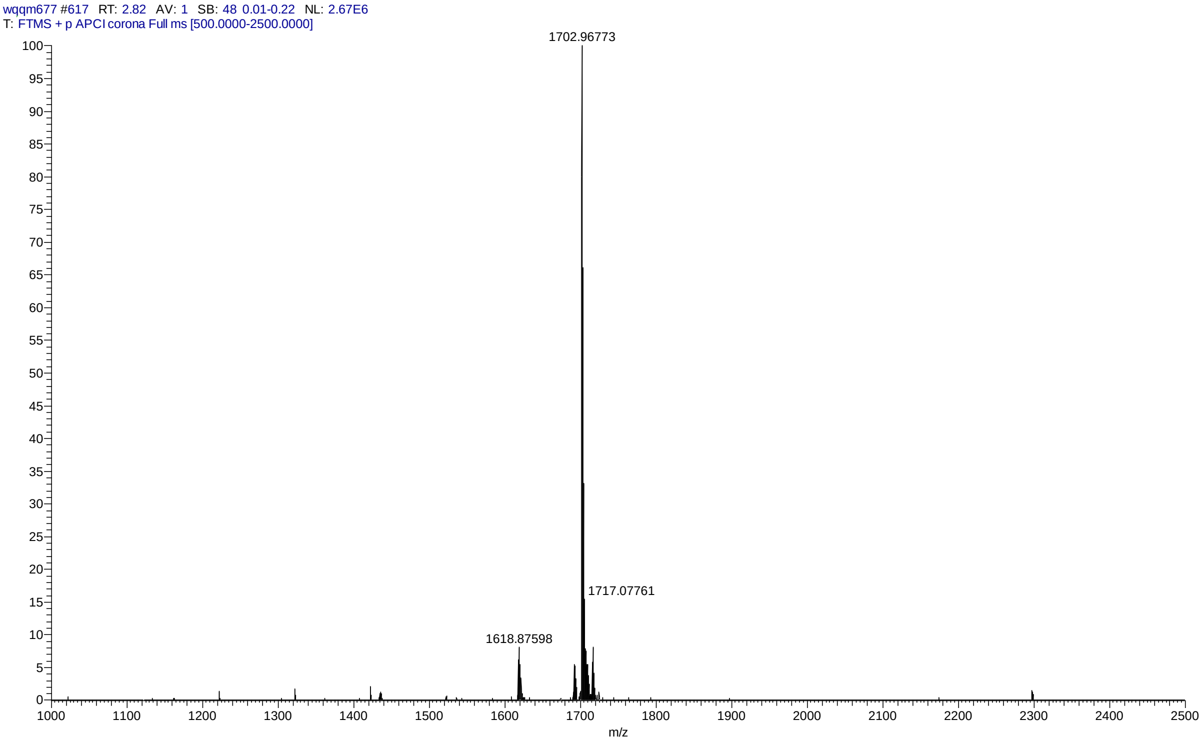

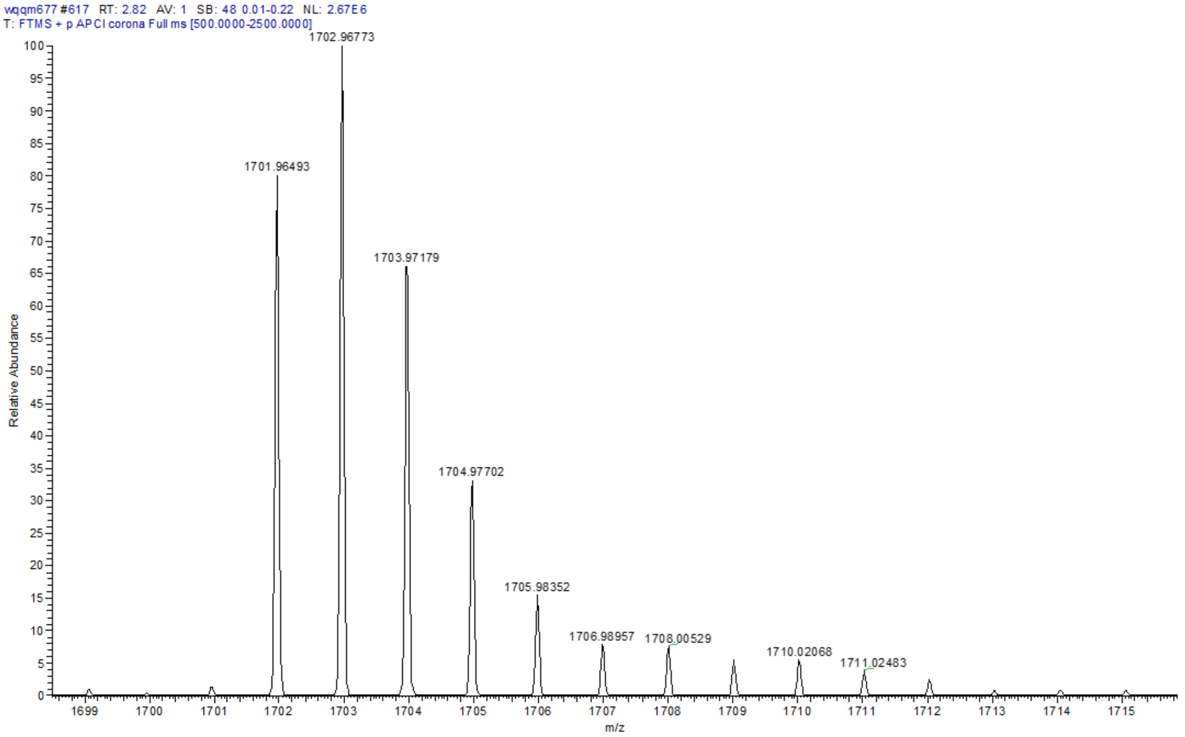

Figure S5 High resolution mass spectra of **3**, HRMS (APCI): calcd. for $C_{119}H_{140}O_9$ ([M+H]$^+$): 1701.96311; found: 1701.96493.

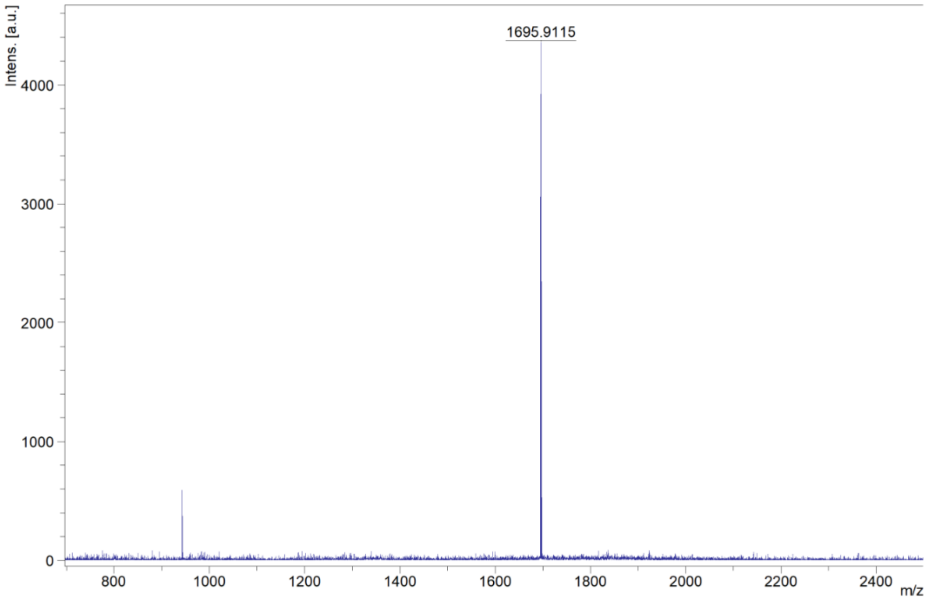

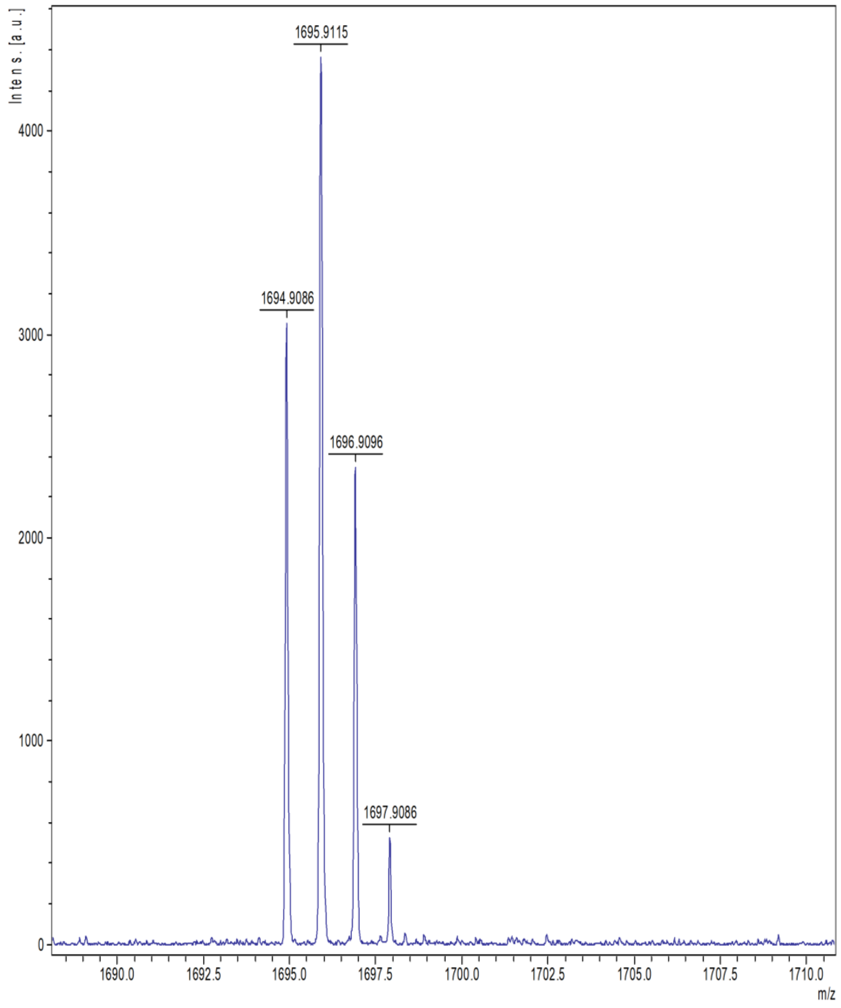

Figure S6 High resolution mass spectra of **11**, HRMS (APCI): calcd. for $C_{119}H_{122}O_9$ ($[M+H]^+$): 1695.9117; found: 1695.9115.

7. NMR spectra

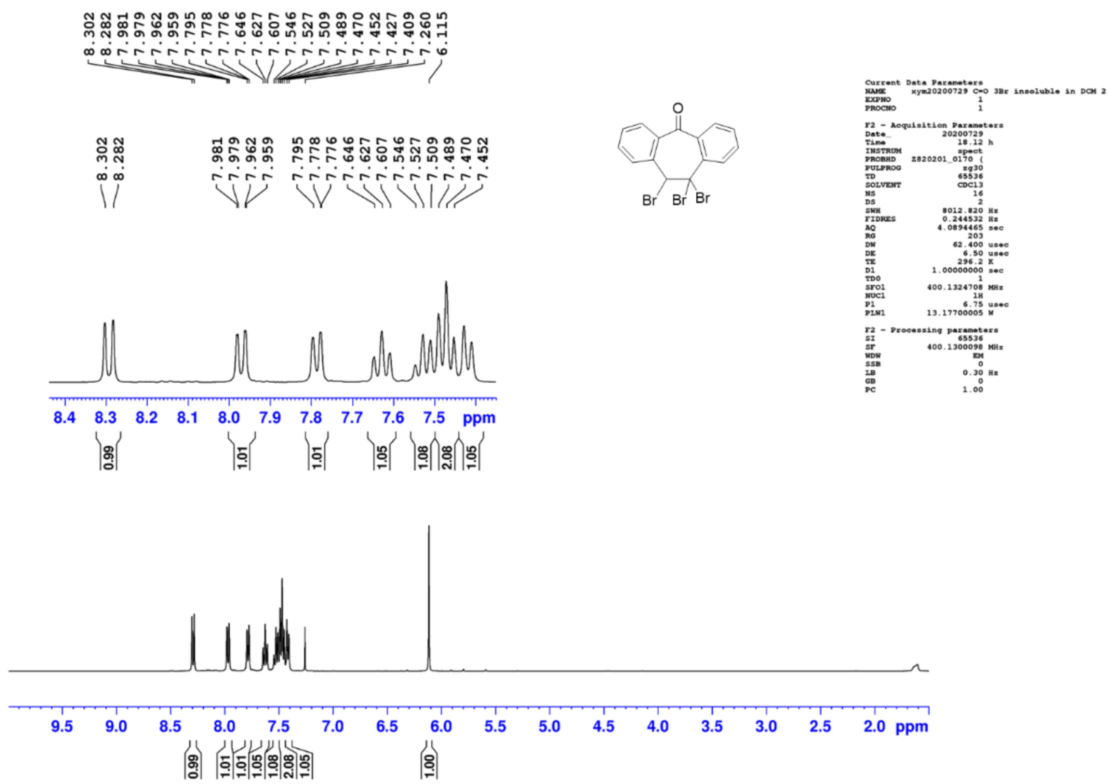

Figure S7 ^{1}H NMR spectrum of **5** in $CDCl_3$.

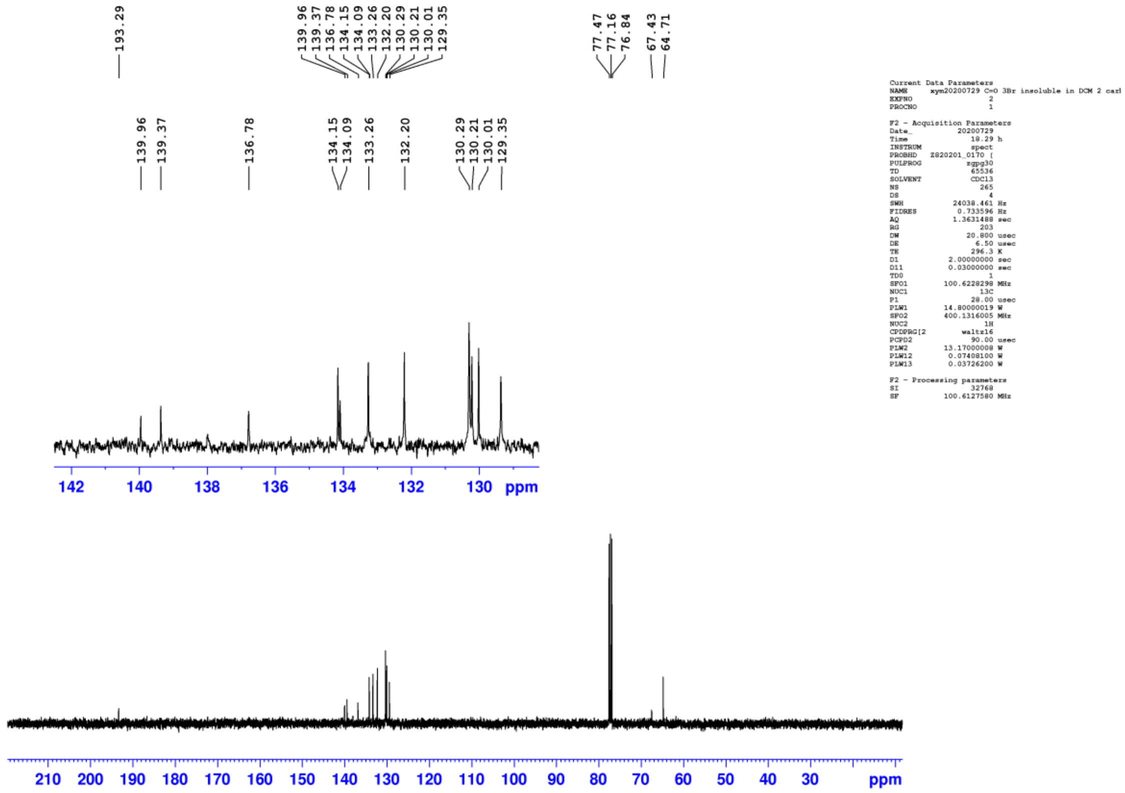

Figure S8 ^{13}C NMR spectrum of **5** in $CDCl_3$.

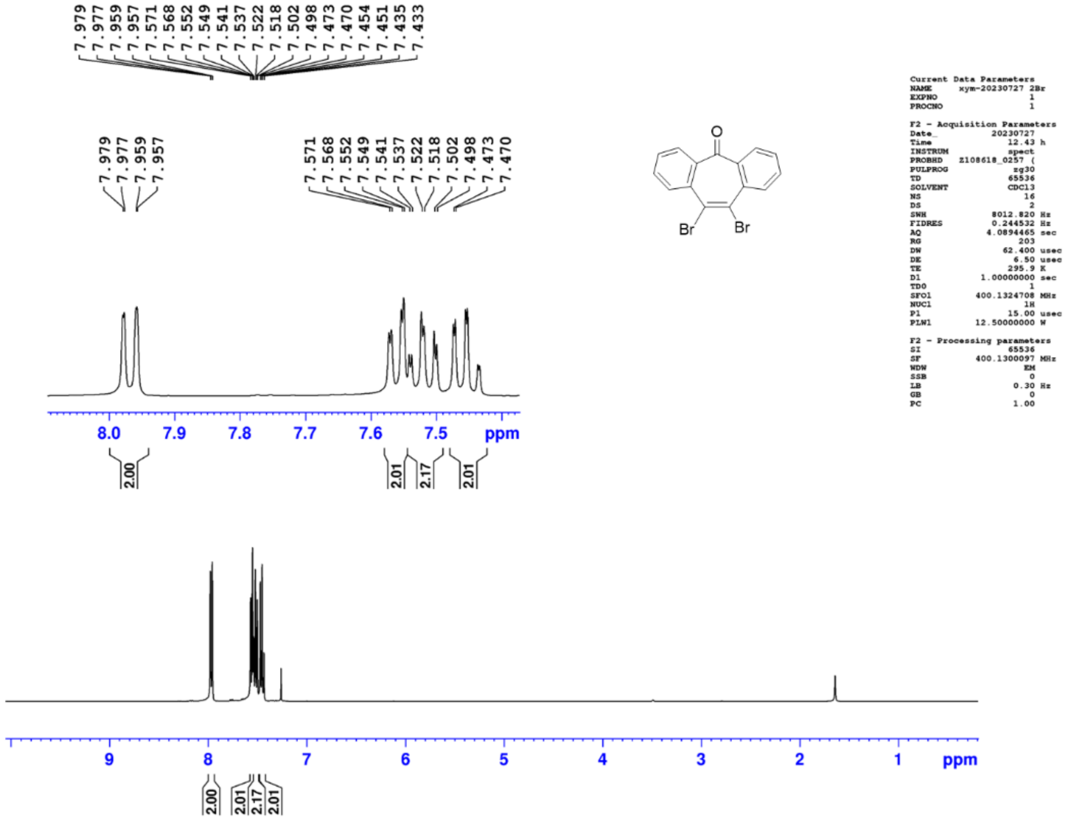

Figure S9 ^{1}H NMR spectrum of **6** in $CDCl_3$.

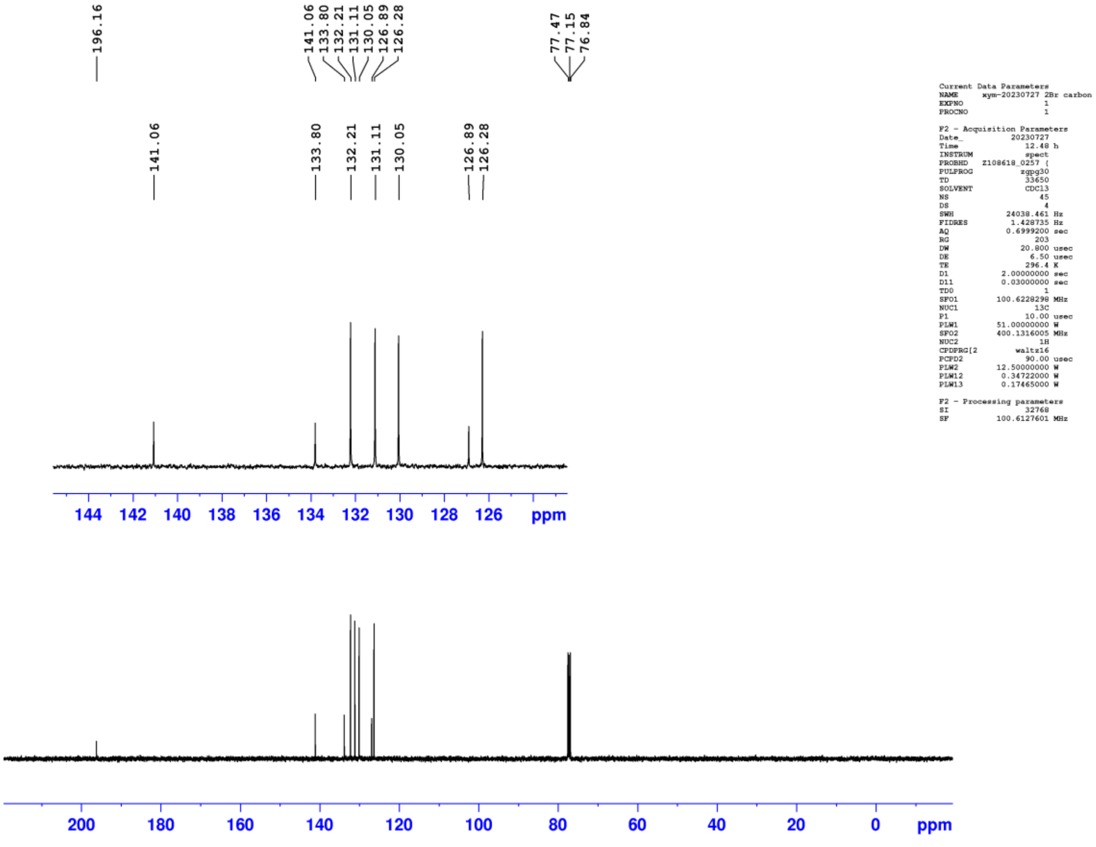

Figure S10 ^{13}C NMR spectrum of **6** in $CDCl_3$.

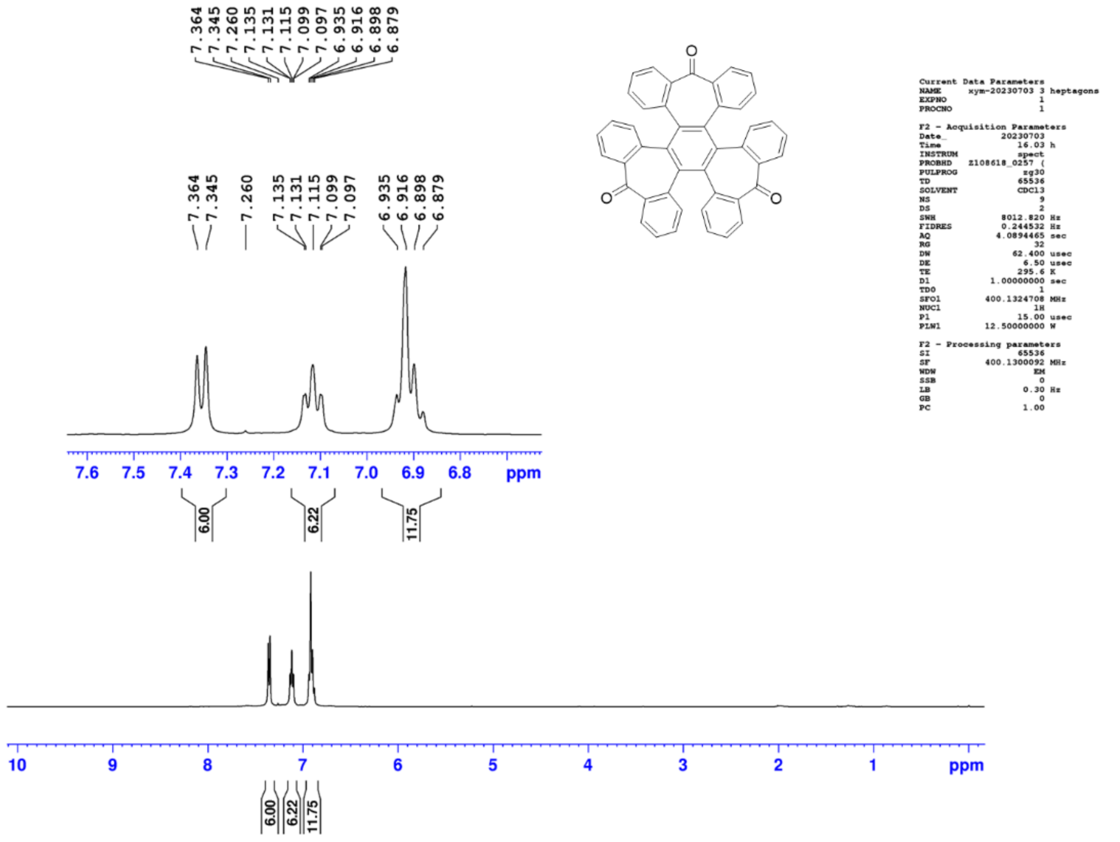

Figure S11 ^{1}H NMR spectrum of **1** in $CDCl_3$

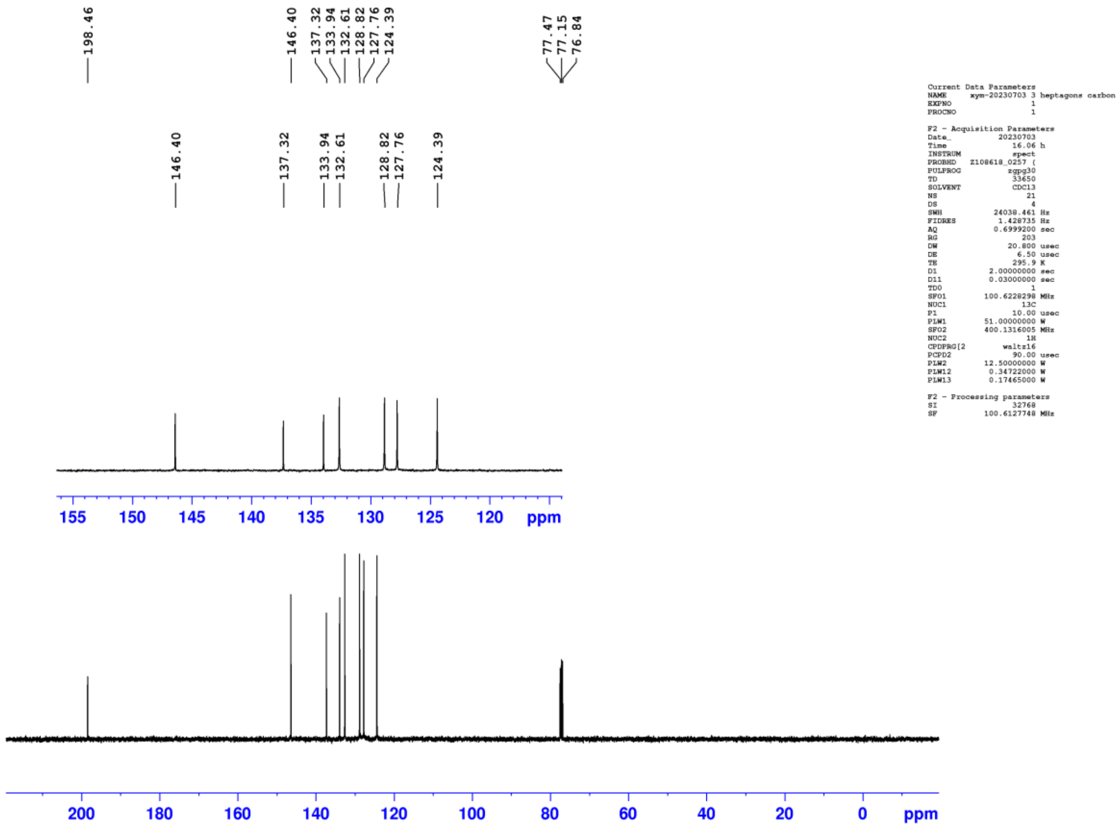

Figure S12 ^{13}C NMR spectrum of **1** in $CDCl_3$

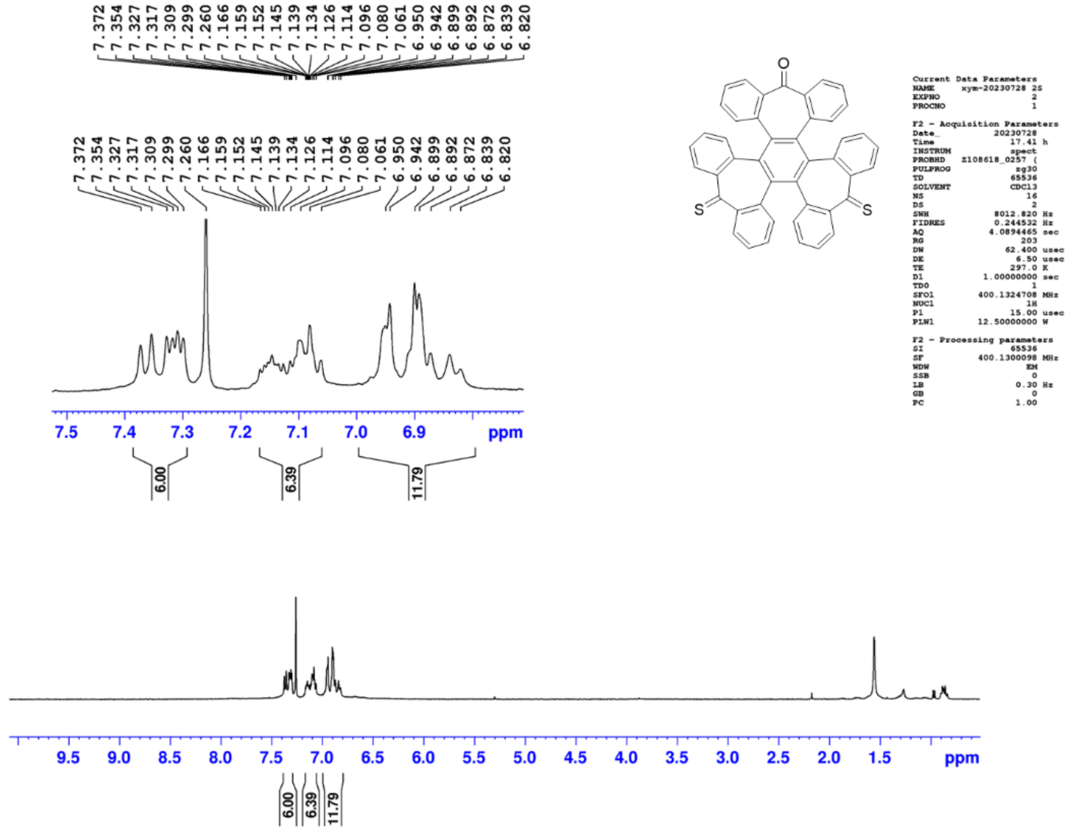

Figure S13 ^{1}H NMR spectrum of **8a** in $CDCl_3$

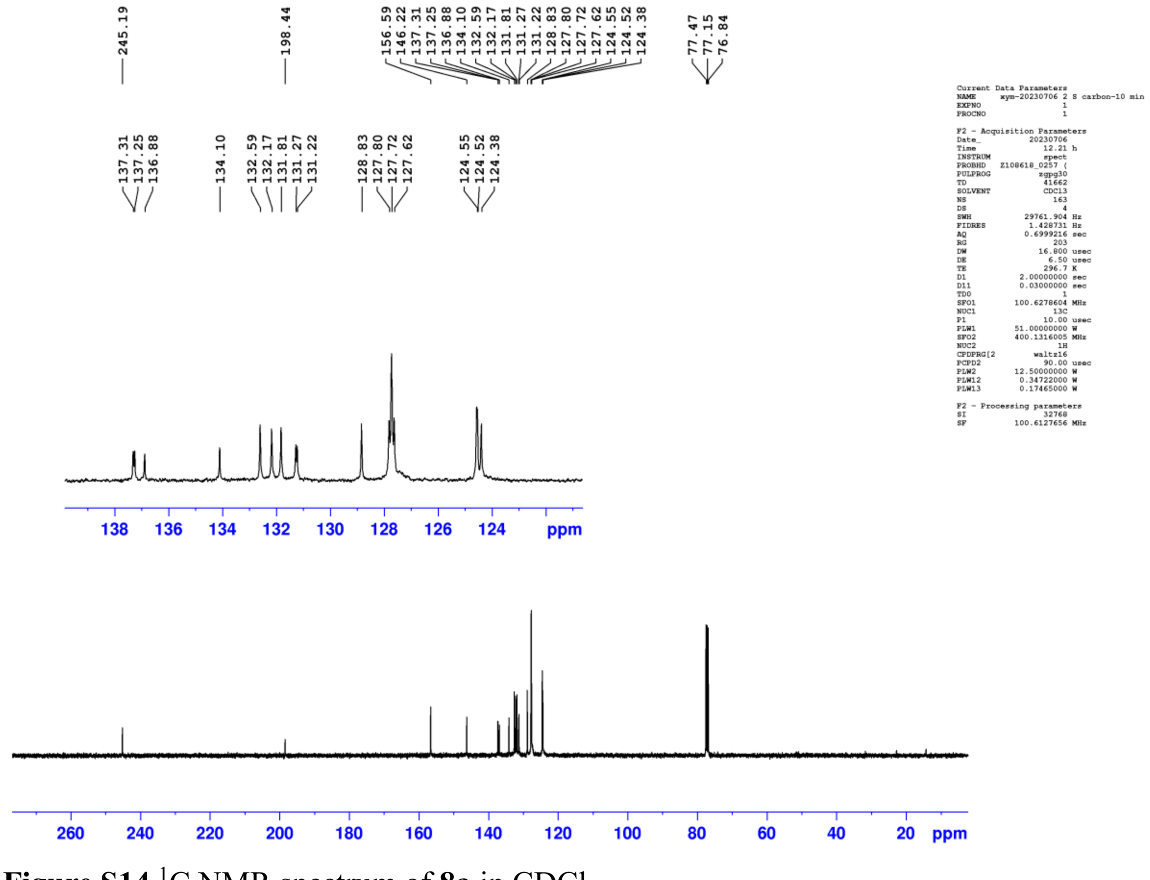

Figure S14 [1]C NMR spectrum of **8a** in $CDCl_3$

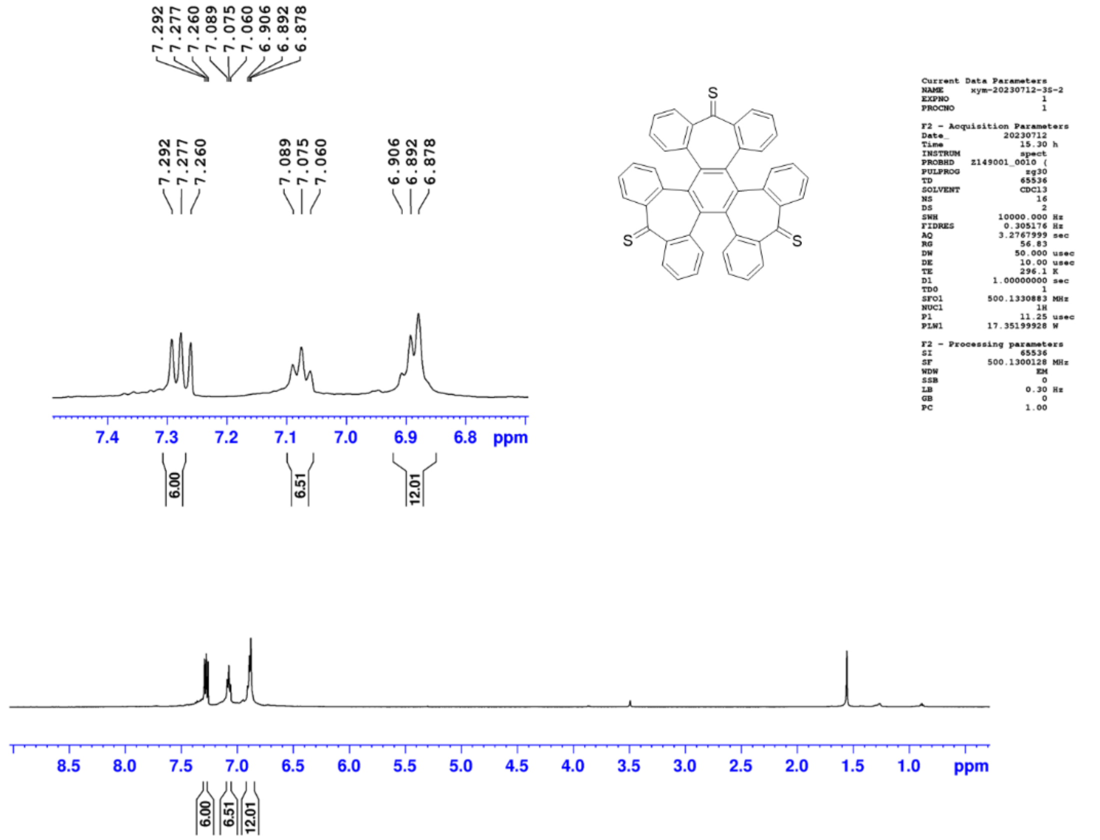

Figure S15 ^{1}H NMR spectrum of **8b** in $CDCl_3$.

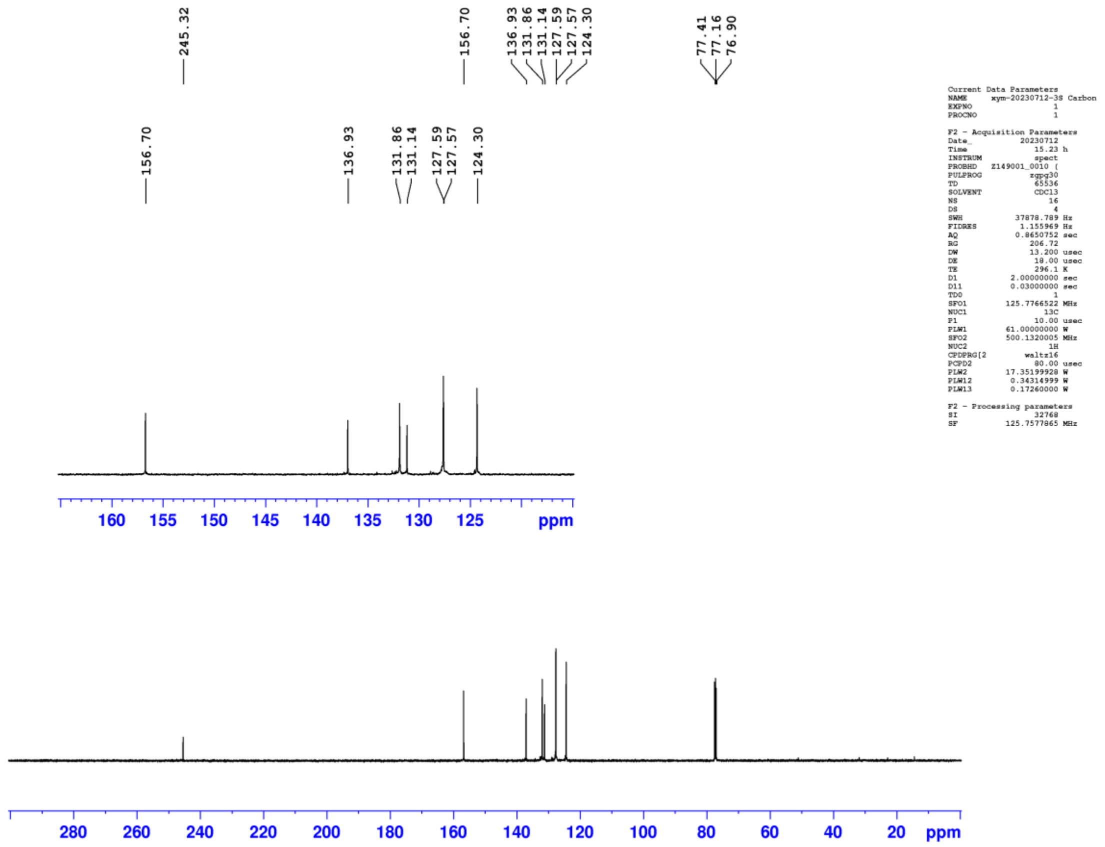

Figure S16 ^{13}C NMR spectrum of **8b** in $CDCl_3$

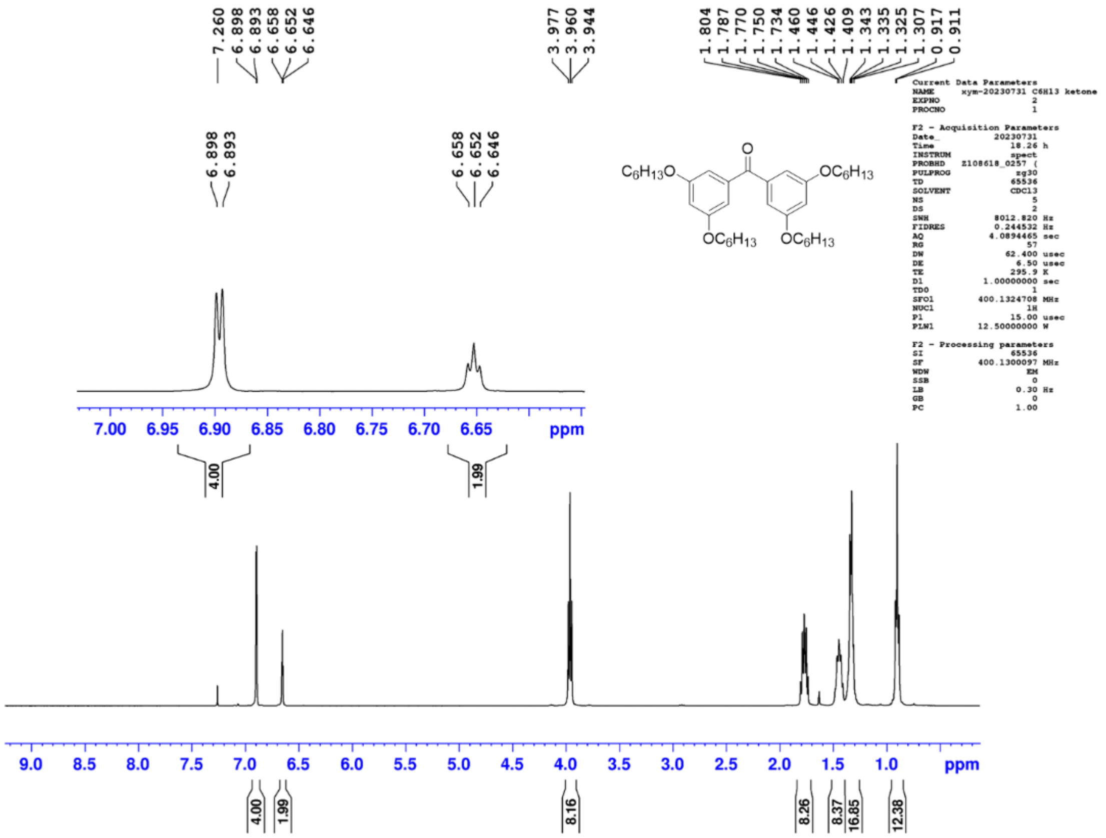

Figure S17 ^{1}H NMR spectrum of **S2** in $CDCl_3$

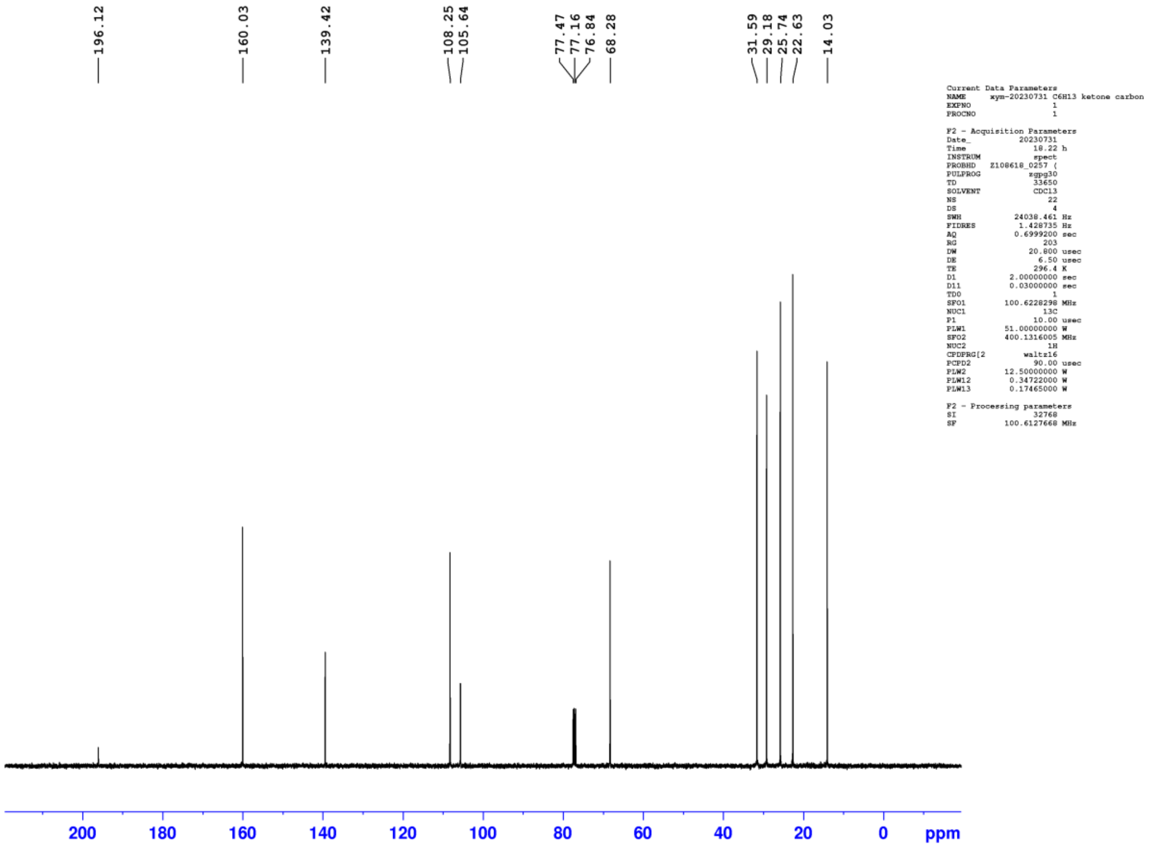

Figure S18 ^{13}C NMR spectrum of **S2** in $CDCl_3$

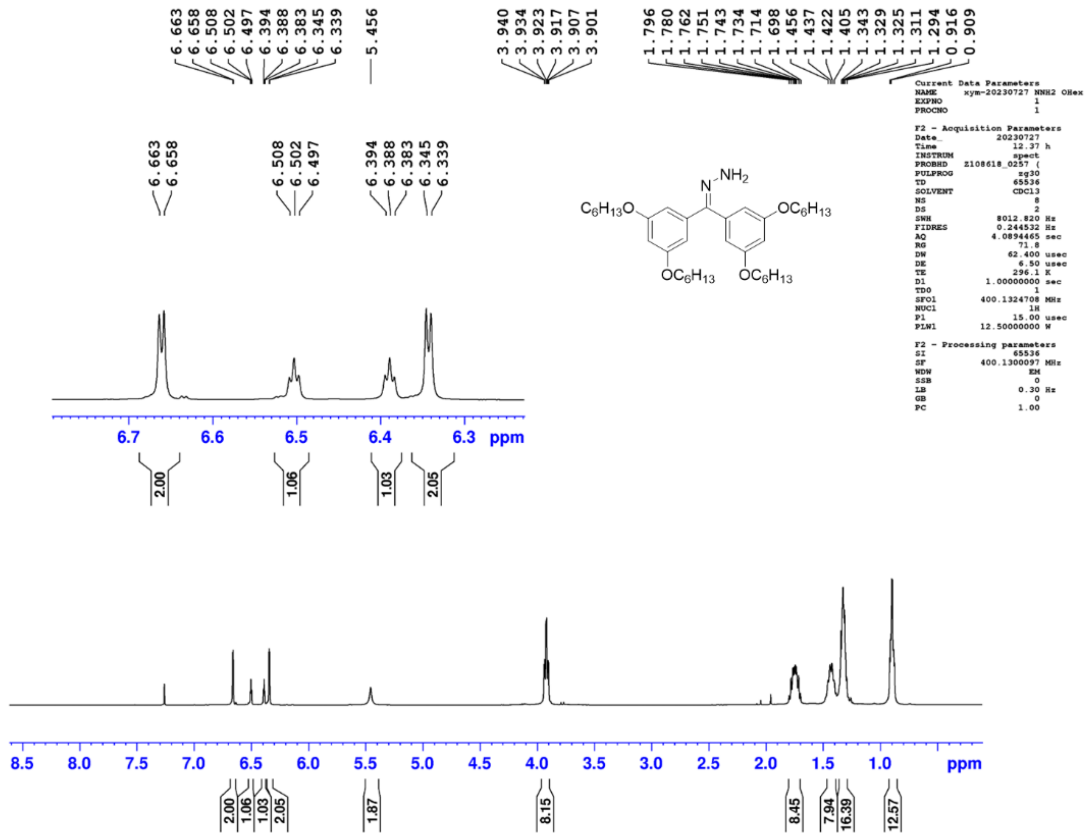

Figure S19 ^{1}H NMR spectrum of **S3** in $CDCl_3$

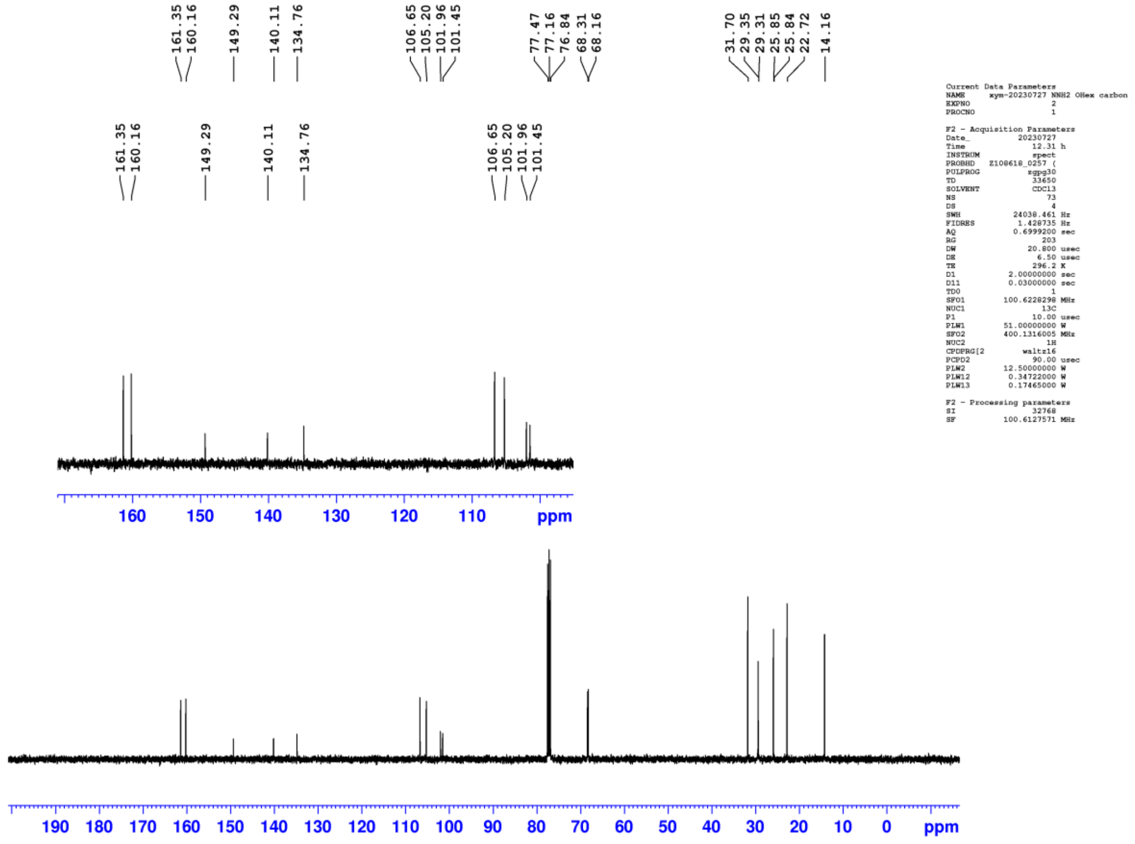

Figure S20 ^{13}C NMR spectrum of **S3** in $CDCl_3$

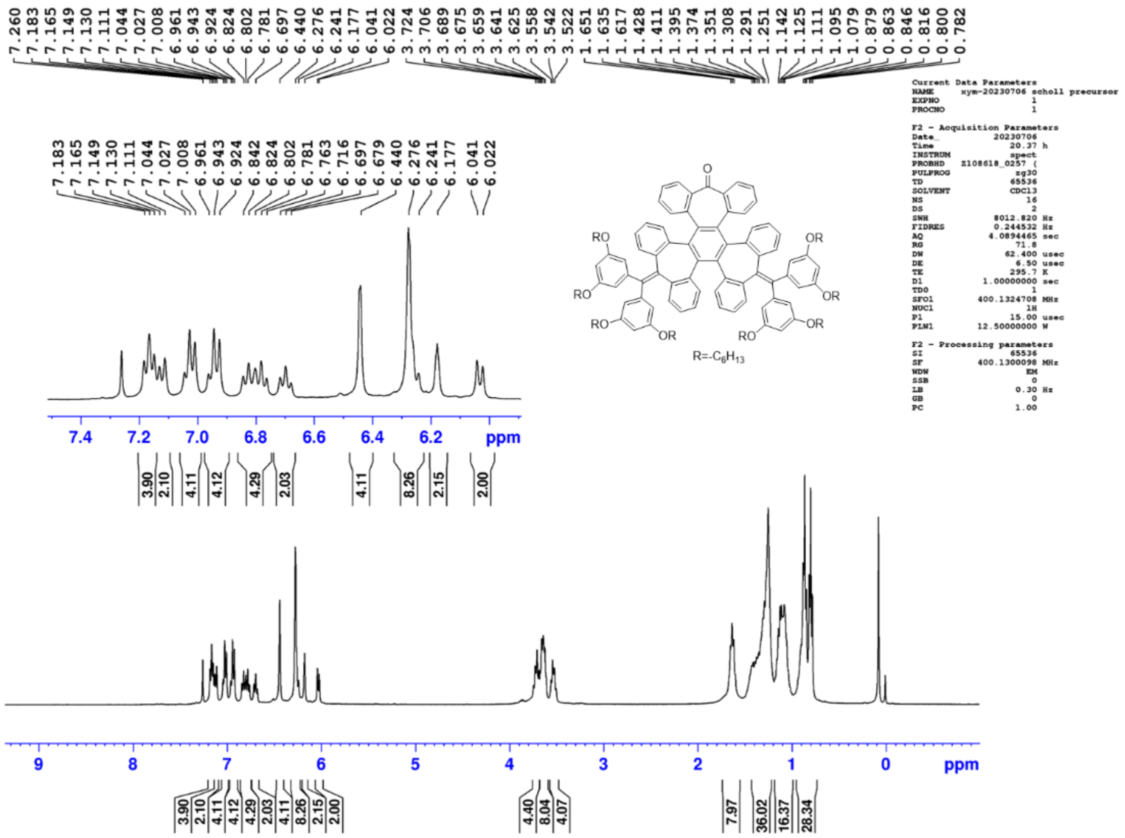

Figure S21 ^{1}H NMR spectrum of **10** in $CDCl_3$

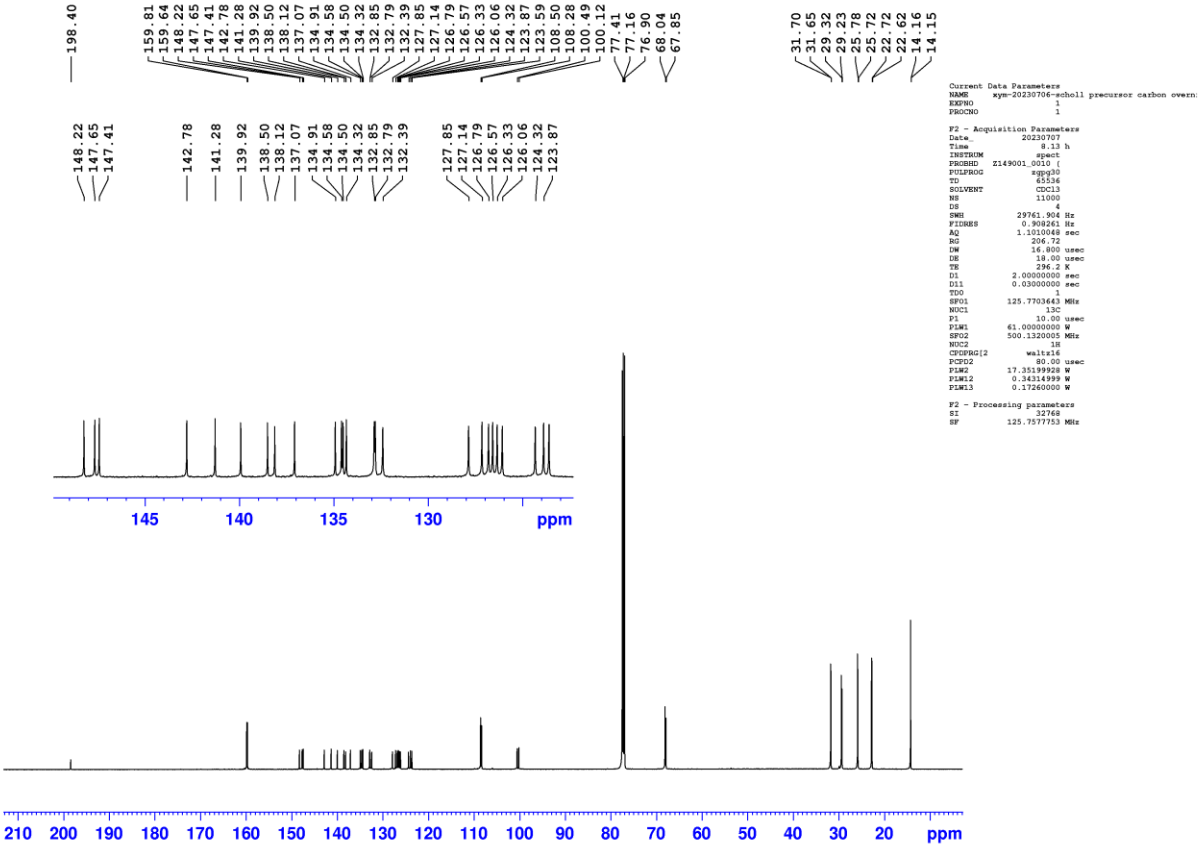

Figure S22 ^{13}C NMR spectrum of **10** in $CDCl_3$

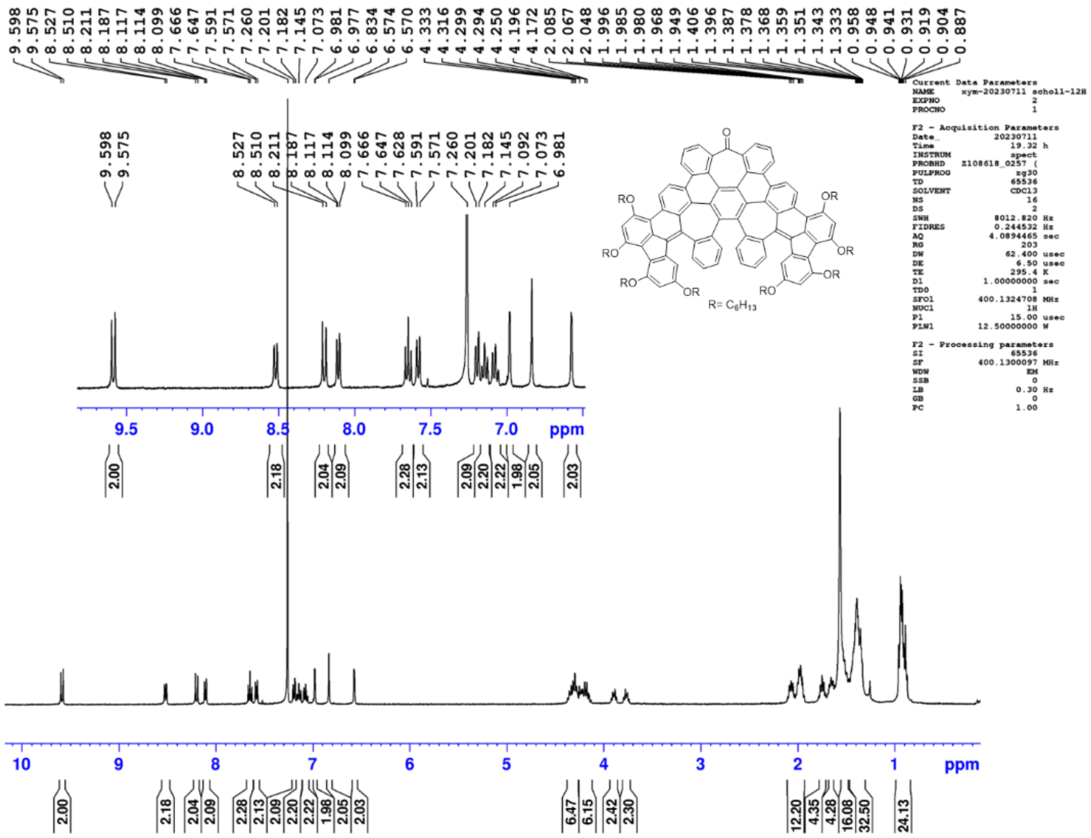

Figure S23 ^{1}H NMR spectrum of **3** in $CDCl_3$

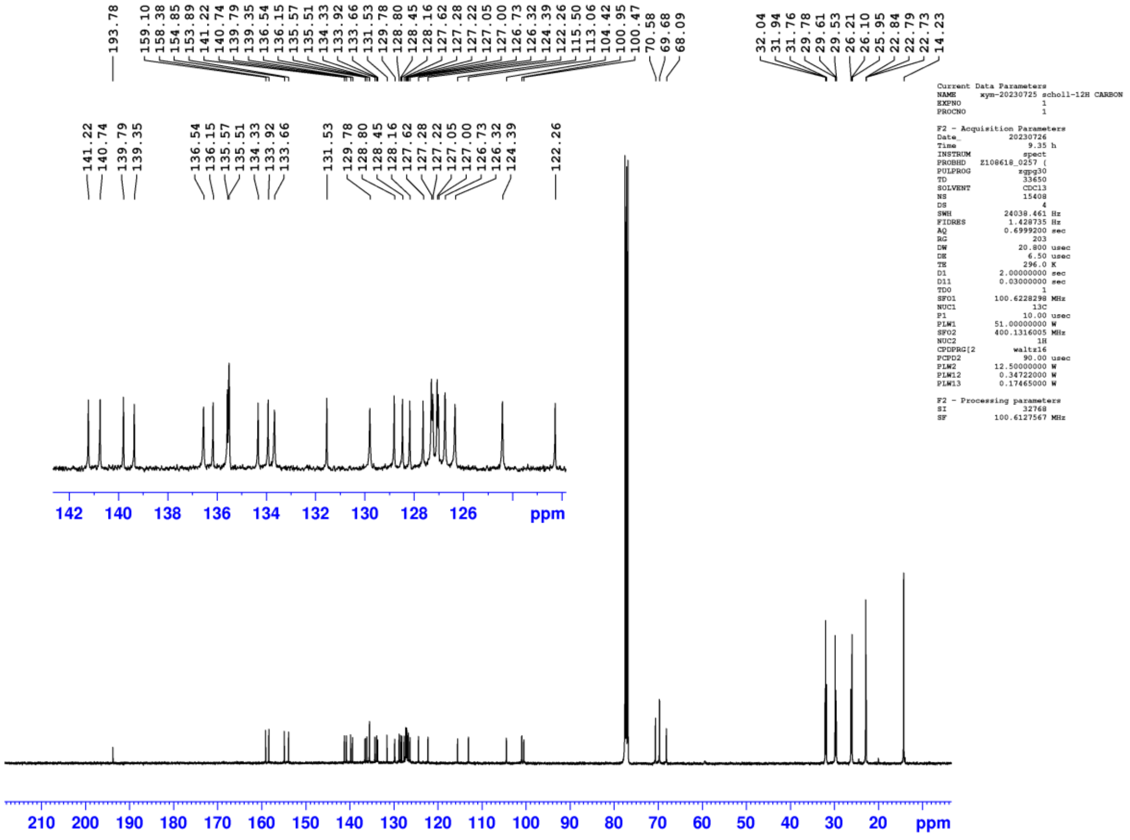

Figure S24 ^{13}C NMR spectrum of **3** in $CDCl_3$

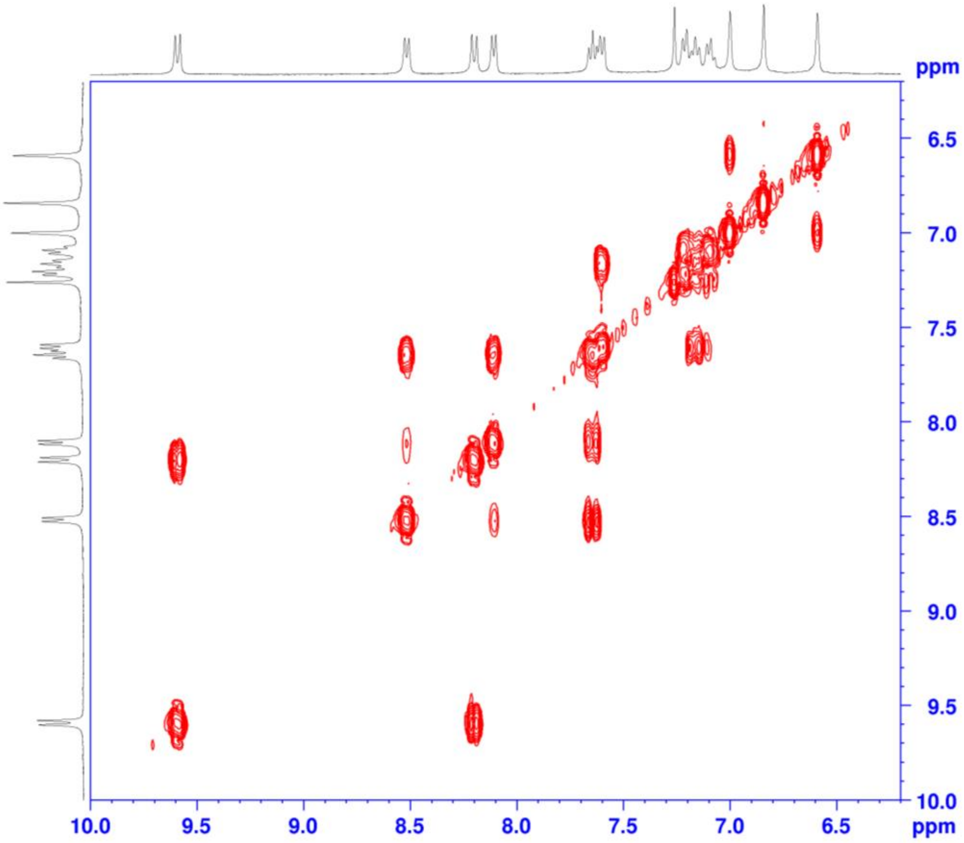

Figure S25 COSY spectrum of **3** in $CDCl_3$. (Aromatic region.)

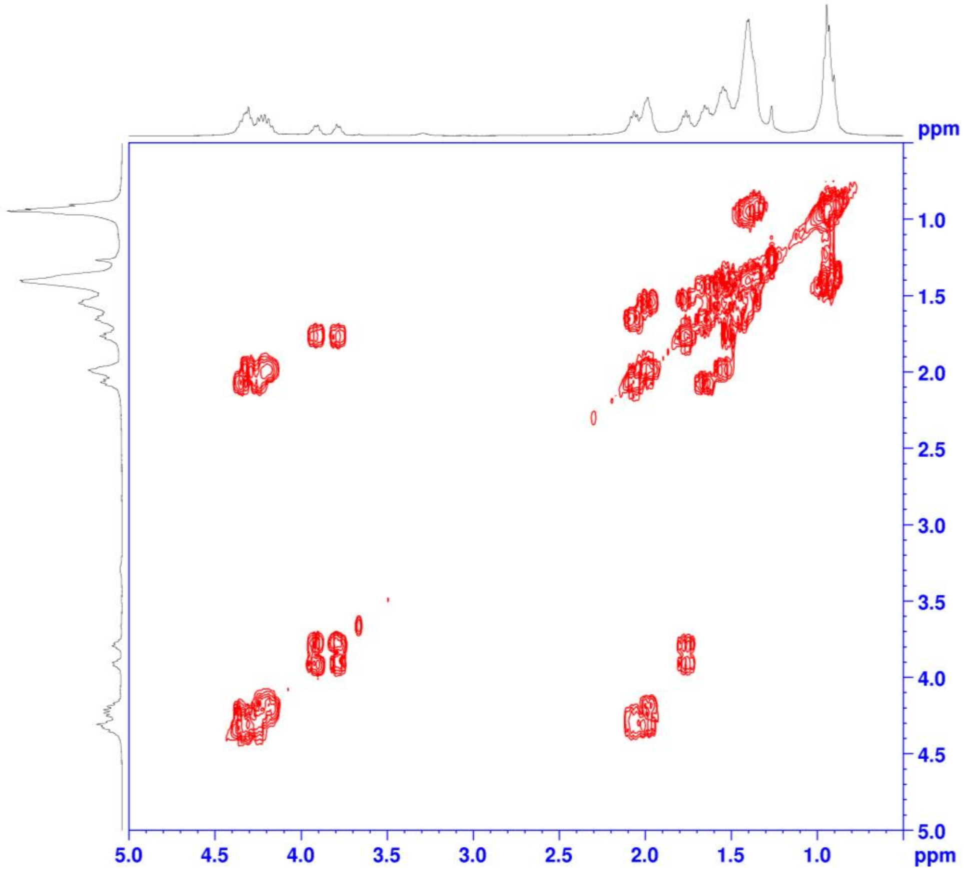

Figure S26 COSY spectrum of **3** in $CDCl_3$. (Aliphatic region.)

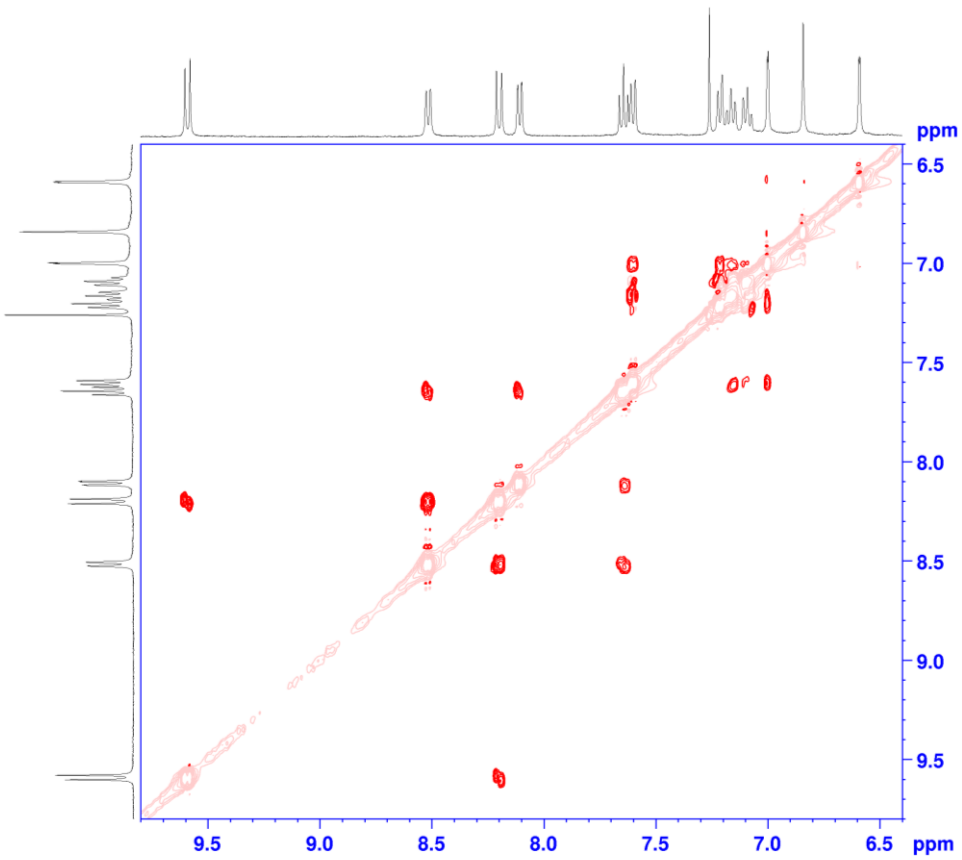

Figure S27 ROESY spectrum of **3** in $CDCl_3$. (Aromatic region.)

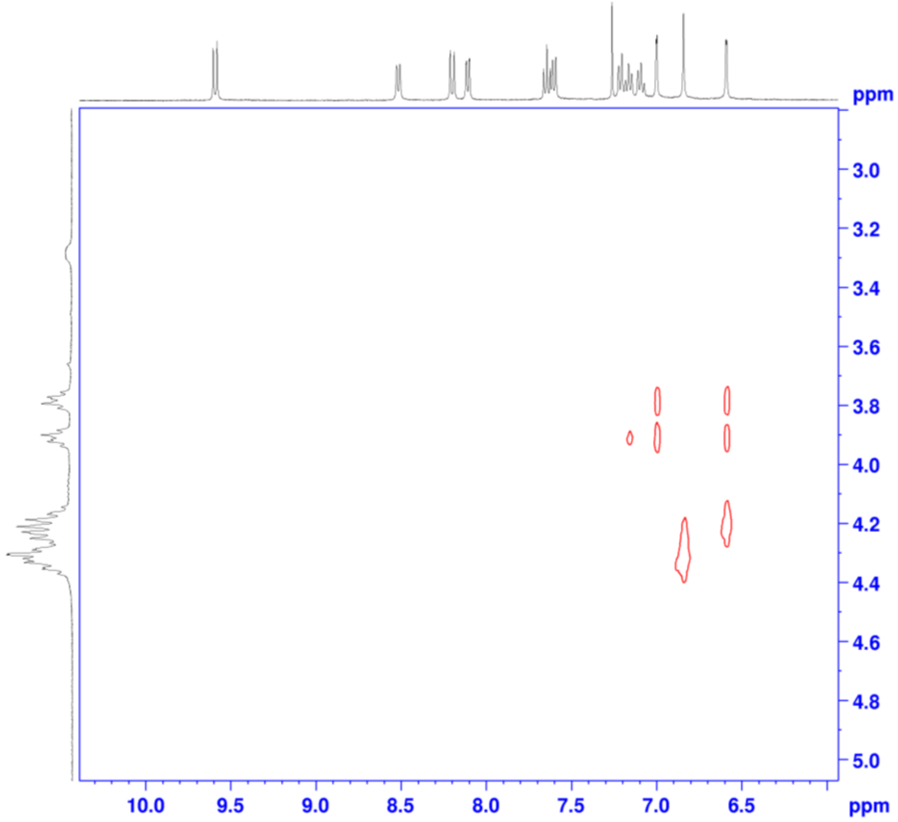

Figure S28 ROESY spectrum of **3** in $CDCl_3$. (Aromatic region versus aliphatic region.)

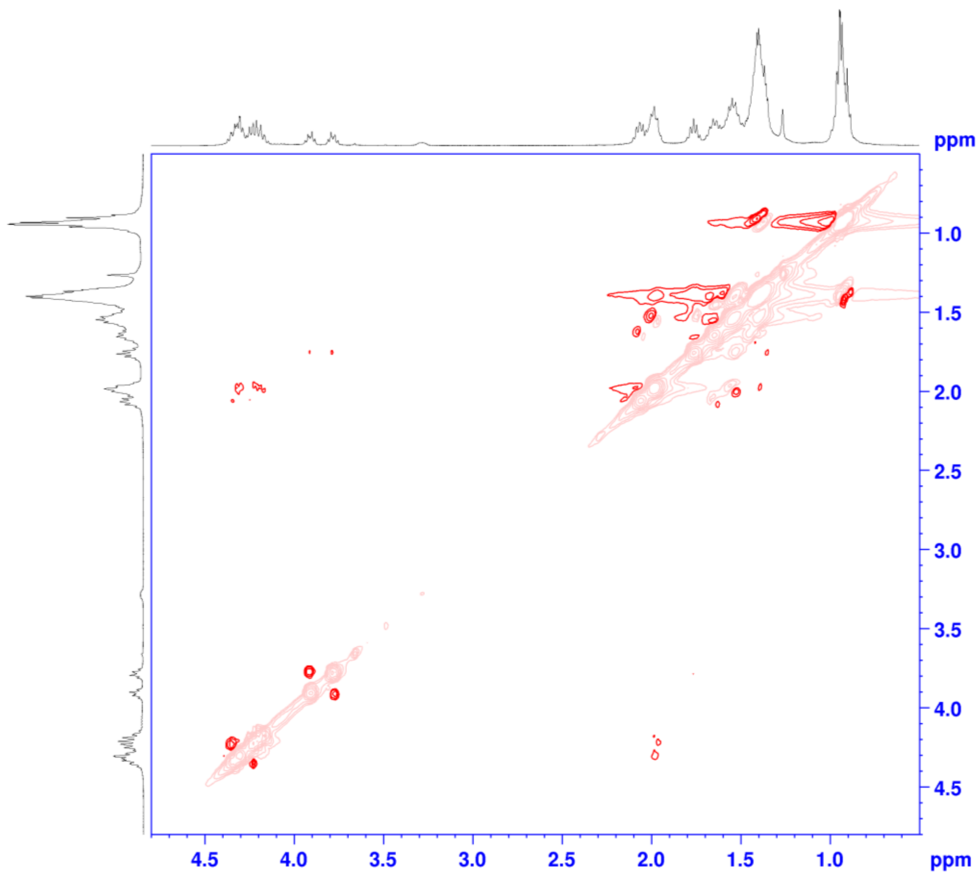

Figure S29 ROESY spectrum of **3** in $CDCl_3$. (Aliphatic region.)

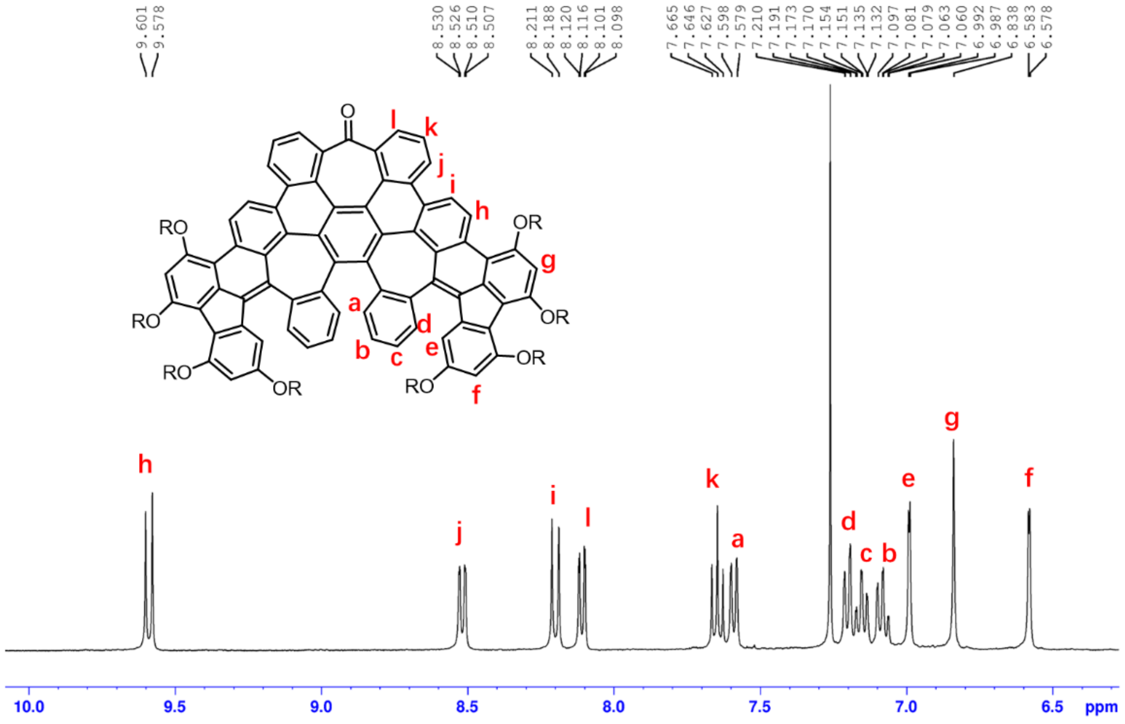

Figure S30 Assignment of H signals of **3**.

8. References

(1) Rupard, J. H.; De Paulis, T.; Janowsky, A.; Smith, H. E. *J. Med. Chem.* **1989**, *32*, 2261–2268.

(2) Cameron, J. F.; Willson, C. G.; Fréchet, J. M. J. *J. Am. Chem. Soc.* **1996**, *118*, 12925–12937.

(3) Berlman, I. *Handbook of Fluorescence Spectra of Aromatic Molecules*, 2nd ed.; Academic Press, 1971.

(4) Gaussian 16 Rev. C.02; Frisch, M. J.; Trucks, G. W.; Schlegel, H. B.; Scuseria, G. E.; Robb, M. A.; Cheeseman, J. R.; Scalmani, G.; Barone, V.; Petersson, G. A.; Nakatsuji, H.; Li, X.; Caricato, M.; Marenich, A. V.; Bloino, J.; Janesko, B. G.; Gomperts, R.; Mennucci, B.; Hratchian, H. P.; Ortiz, J. V.; Izmaylov, A. F.; Sonnenberg, J. L.; Williams; Ding, F.; Lipparini, F.; Egidi, F.; Goings, J.; Peng, B.; Petrone, A.; Henderson, T.; Ranasinghe, D.; Zakrzewski, V. G.; Gao, J.; Rega, N.; Zheng, G.; Liang, W.; Hada, M.; Ehara, M.; Toyota, K.; Fukuda, R.; Hasegawa, J.; Ishida, M.; Nakajima, T.; Honda, Y.; Kitao, O.; Nakai, H.; Vreven, T.; Throssell, K.; Montgomery Jr., J. A.; Peralta, J. E.; Ogliaro, F.; Bearpark, M. J.; Heyd, J. J.; Brothers, E. N.; Kudin, K. N.; Staroverov, V. N.; Keith, T. A.; Kobayashi, R.; Normand, J.; Raghavachari, K.; Rendell, A. P.; Burant, J. C.; Iyengar, S. S.; Tomasi, J.; Cossi, M.; Millam, J. M.; Klene, M.; Adamo, C.; Cammi, R.; Ochterski, J. W.; Martin, R. L.; Morokuma, K.; Farkas, O.; Foresman, J. B.; Fox, D. J.: Wallingford, CT, 2019.
